# Dietary supplementation of *Lycium barbarum* polysaccharides alleviates soybean meal-induced enteritis in spotted sea bass *Lateolabrax maculatus*

**DOI:** 10.1016/j.aninu.2024.10.005

**Published:** 2024-11-15

**Authors:** Longhui Liu, Yanbo Zhao, Zhangfan Huang, Zhongying Long, Huihui Qin, Hao Lin, Sishun Zhou, Lumin Kong, Jianrong Ma, Yi Lin, Zhongbao Li

**Affiliations:** a Fisheries College, Jimei University, Xiamen, China; b Fujian Provincial Key Laboratory of Marine Fishery Resources and Eco-environment, Xiamen, China

**Keywords:** *Lycium barbarum* polysaccharides, Spotted sea bass, Intestinal inflammation, Transcriptomics and metabolomics

## Abstract

The aim of this experiment was to investigate the effect of *Lycium barbarum* polysaccharides (LBP) on alleviating soybean meal-induced enteritis (SBMIE) in spotted sea bass *Lateolabrax maculatus*. The diet with 44% fishmeal (FM) content was used as a blank control, and soybean meal (SM) was used to replace 50% FM as an experimental control to induce enteritis. Then, on the basis of experimental control, 0.10%, 0.15%, and 0.20% LBP were added as experimental diets. A total of 225 spotted sea bass (44.52 ± 0.24 g) were randomly divided into 5 groups and fed the corresponding diets for 52 d. The results showed that 0.15% LBP decreased serum D-lactic acid (D-LA) content and diamine oxidase (DAO) activity (*P* < 0.05). In addition, in all LBP supplementation groups, the intestinal tissue morphology was significantly improved (*P* < 0.05); the intestinal microbial structure gradually recovered to a level close to that without adding SM; and the microbial species richness and diversity were significantly increased (*P* < 0.05). Through transcriptomic and metabolomic analysis, it was found that the expression of proinflammatory factors such as interleukin-1β (*IL-1β*), interleukin-12 (*IL-12*), nuclear factor kappa B subunit 2 (*NF-κB2*), and Toll-like receptor 2 (*TLR2*) were significantly down-regulated in the mitogen-activated protein kinase (MAPK) and Toll-like receptor signaling pathways (*P* < 0.05), and the important tight junction protein gene *Occludin* was up-regulated (*P* < 0.05). In addition, LBP down-regulated saponin metabolites and up-regulated amino acid metabolites (*P* < 0.05). In conclusion, LBP demonstrated a significant alleviating effect on SBMIE of spotted sea bass *L. maculatus.*

## 1 Introduction

Aquatic products are critical to achieving the goal of food and nutrition security, and aquaculture is the most important way to ensure an adequate supply of aquatic products (World Health Organization, 2018; Willett et al., 2019). In the last 30 years, aquaculture has produced about half of all aquatic products consumed by humans, particularly fish, which provides at least 15% of the per capita animal protein intake for more than 4.5 billion people (Bene et al., 2015; Gephart et al., 2020). On the other hand, aquaculture is the largest consumer of fishmeal (FM), which has been considered the main protein source in aquafeed because it has a very high digestible protein content and balanced amino acid profile (Gatlin et al., 2007; Tran et al., 2022). However, the sharp decrease in the quantity of annual fish caught to make FM has meant that the price of FM has more than doubled over the past 20 years. It is therefore not sustainable for aquaculture to be dependent on FM to provide high-quality protein. Currently, a sustainable feed solution for aquaculture is being adopted—renewable plant-sourced protein to replace FM (Naylor et al., 2009, 2021). Soybean meal (SM) is an important vegetable protein source for aquatic feed in the world because of its high protein, relatively balanced amino acid composition and low price (Herman and Schmidt, 2016; Jia et al., 2022; Trushenski et al., 2006). Unfortunately, anti-nutritional factors (ANFs) such as soybean antigen, trypsin inhibitor, α-amylase inhibiting factor, lectin, and phytic acid were detected in SM (Francis et al., 2001; Gatlin et al., 2007; Liener, 1994). It is well known that ANFs not only reduce food intake, palatability, and digestive enzyme activity but also further destroy the integrity of the intestinal mucosa, thereby inducing intestinal inflammation (Arriaga-Hernández et al., 2021; Herman and Schmidt, 2016; Rossi et al., 2021). The intestinal inflammation caused by the addition of SM is called soybean meal-induced enteritis (SBMIE) and is known as non-infectious subacute enteritis in fish. Intestinal histomorphology of fish with SBMIE is usually accompanied by shortening of mucosal fold height, swelling of lamina propria and submucosa, infiltration of various inflammatory cells, and decreased absorption vacuoles of intestinal epithelial cells. Furthermore, the dysregulation of the expression of tight junction and inflammation genes such as zonula occluden-1 (*ZO1*) and interleukin-10 (*IL-10*), which are involved in the development of SBMIE (Gu et al., 2018; Ke et al., 2022; Wang et al., 2022; Zhang et al., 2021). However, it has been conclusively demonstrated that sensitivity to ANFs varies among various fish species receiving identical quantities of FM and SM. Rainbow trout (*Oncorhynchus mykiss*) and Atlantic salmon (*Salmo salar*) (Refstie et al., 2000) showed morphological alternations in the intestine, while no distinct adverse effects could be reported in Japanese flounder (*Paralichthys olivaceus*) (Kikuchi, 1999) and red drum (*Sciaenops ocellatus*) (Reigh and Ellis, 1992). It is worth mentioning that these symptoms, especially intestinal inflammation, are more pronounced in carnivorous fish (Gu et al., 2016; Laporte and Trushenski, 2012; Wang et al., 2021).

Prebiotics are indigestible carbohydrates that can be classified according to degree of polymerization (number of monosaccharide units) or molecular size, such as polysaccharides and monosaccharides (Akhter et al., 2015). Prebiotics have been reported to repair SBMIE through a series of modulatory effects on the intestinal tract (Burr et al., 2010; Sealey et al., 2015), but most of the above reports on relieving SBMIE have focused on the use of monosaccharides. By contrast, the effects of polysaccharides have rarely been reported; however, as a class of prebiotics, polysaccharides may also have the potential to repair SBMIE. Polysaccharides are abundant in nature and come from a variety of sources such as plants, fruits, algae, and bacteria. They have similar biological activity, including anti-inflammatory properties, antioxidant capacity enhancement, and the ability to regulate the intestinal microbiota, which have been reported in previous studies (Do et al., 2021; Liu et al., 2018; Tang et al., 2020; Sousa et al., 2018). In fact, polysaccharides have been reported to repair intestinal inflammation in mice, rats, and some terrestrial animals. For example, Li et al., 2022a, Li et al., 2022b reported that pine pollen polysaccharides and sulfated polysaccharides could protect mice with ulcerative colitis from inflammation by regulating the receptor-interacting protein kinase 3 (RIPK3)-dependent necroptosis pathways and the tight junction of colonic epithelial cells. Shen et al. (2023) reported that the mixture of seaweed polysaccharide and tea polyphenols was effective in inhibiting the production of interleukin-6 (IL-6) and tumor necrosis factor α (TNF-α) in RAW264.7 cells induced by lipopolysaccharide. Notably, Hou et al. (2020) reviewed recent reports of polysaccharides with anti-inflammatory effects, summarizing the various pharmacological mechanisms and targets involved and discussing structure–activity relationships in detail. These reviews and studies further indicated the possibility of polysaccharides to repair intestinal inflammation in aquaculture and provided a reference for future studies. Limited but valuable studies have reported that polysaccharides can obviously improve the intestinal barrier, immune response, and reduce intestinal inflammation in aquatic animals such as rainbow trout (*O. mykiss*) (Deng et al., 2021), tilapia (*Oreochromis niloticus*) (Liu et al., 2022), turbot (*Scophthalmus maximus*) (Fuchs et al., 2015), and so on. Polysaccharides have therefore been found to exert beneficial effects on the intestinal tract, which make it possible to repair the damage caused by SBMIE (Liu et al., 2021; Zhu et al., 2021a).

*Lycium barbarum* is a traditional Chinese medicine, and it has also been called Goji in China. *L. barbarum* has been used as a common health product and medicine in Asia for thousands of years and contains a variety of phytochemicals and nutrients, including caffeic acid, rutin, p-coumaric acid, and so on, but its most important and abundant functional component is *L. barbarum* polysaccharides (LBP) (Amagase and Farnsworth, 2011; Chang et al., 2019). In the previous review, the antioxidant, immune regulatory, and anti-inflammatory effects of LBP were summarized (Tian et al., 2019). Sun et al. (2023) summarized the prebiotic effects of *L. barbarum* and suggested that LBP could prevent inflammatory bowel disease (IBD) through the growth of the intestinal microbiota and the production of metabolites, especially bile acids and short-chain fatty acids (SCFAs). Moreover, recent studies have shown that the anti-inflammatory effect of LBP is due to inhibiting the expression of proinflammatory factors, mitogen-activated protein kinase (MAPK)-mediated inflammatory cell signaling pathways, and nuclear factor kappa B (NF-κB) (Jin et al., 2013). LBP has been shown to have a great anti-inflammatory effect, especially in the treatment of IBD, but this has not been reported in relation to SBMIE. LBP may have great potential for mitigating SBMIE and is worthy of further study.

Spotted sea bass (*Lateolabrax maculatus*) is a kind of commercial fish widely cultured in the coastal areas of China because of its delicious meat, rich nutrition, and strong adaptability. The yield of spotted sea bass in 2021 was 199,100 tons, which accounts for 10.80% of China's mariculture fish (Chinese Fisheries Bureau of Ministry of Agriculture, 2022). Spotted sea bass is a typical carnivorous fish, and the symptoms of SBMIE are quite severe, especially in intestinal nutrient transport (intestinal mucosal barrier) and expression of inflammatory factors (Zhang et al., 2018). In addition, the price of high-quality Peruvian fish meal is close to 20,000 RMB/ton at its highest. SM is currently only 3500 RMB per ton, and it costs 300 RMB to add 1 g/kg of LBP to a ton of feed (Chen, 2023; Wang et al., 2022). Therefore, the present study was conducted to evaluate the effect of LBP on the alleviation of SBMIE in spotted sea bass and to explore the possibility of using LBP as a feed additive in aquaculture to reduce the proportion of FM in feed formulation to lower production costs and mitigate the negative effects of SM in fish.

## 2 Materials and methods

### 2.1 Animal ethics statement

This experiment was approved by the Animal Ethics Committee of Jimei University (Grant No. JMU202103009). The author has complied with all relevant ethical laws.

### 2.2 LBP preparation and characterization

This study isolated two compositions from LBP. The yield and molecular weight of composition 1 were 20.9% and 35.508 kDa, respectively, including xylose (0.27%), mannose (0.44%), galactose (1.01%), arabinose (1.69%) and glucose (96.59%). The yield and molecular weight of composition 2 were 31.4% and 11.343 kDa, respectively, including galactose (0.08%), arabinose (0.21%), mannuronic acid (5.19%), guluronic acid (7.04%), glucuronic acid (9.36%), galacturonic acid (16.57%) and glucose (61.55%). The specific methods and results refer to a previous report (Huang et al., 2023a).

### 2.3 Experimental diets

Five groups of feed with crude protein of about 45% and crude fat of about 12% were designed according to the nutritional requirements of spotted sea bass (Ai et al., 2004), including the diet of spotted sea bass that was produced to induce SBMIE by 40% SM content, labeled as the HS group, and the experimental groups supplemented with graded LBP (0.10%, 0.15%, and 0.20%) on the basis of 40% SM content, labeled HL1, HL2, and HL3, respectively. Correspondingly, 44% FM content feed was used as a control and labeled the LS group. Each group was balanced with flour (Table 1).

**Table 1:** Nutritional composition of experimental diets (dry matter basis, %).

Item	Groups				
	LS	HS	HL1	HL2	HL3
Ingredients^1^					
Fish meal	440	220	220	220	220
Soybean meal	0	400	400	400	400
Casein	110	110	110	110	110
Flour	345	149	148	147.5	147
Fish oil	35	50	50	50	50
Soybean oil	25	25	25	25	25
Mineral premix^2^	6	6	6	6	6
Antioxidant	3	3	3	3	3
Ca(H_2_PO_4_)_2_	12	12	12	12	12
Vitamin premix^3^	8	8	8	8	8
Choline	6	6	6	6	6
Methionine	0	1.0	1.0	1.0	1.0
Lecithin	10	10	10	10	10
LBP	0	0	1.0	1.5	2.0
Total	1000	1000	1000	1000	1000
Proximate composition					
Crude protein	42.43	43.52	43.25	43.56	43.61
Crude lipid	11.50	11.70	11.80	11.80	11.80
Crude ash	8.70	8.00	7.80	8.00	8.00
Moisture	7.70	7.00	8.10	7.60	7.60
Calcium	1.79	1.11	1.11	1.1.4	1.21
Phosphorus	1.56	1.26	1.21	1.27	1.20
Gross energy, MJ/kg	18.01	18.19	18.18	18.26	18.02

LBP = *Lycium barbarum* polysaccharides.

1 The proportion of nutrients of the main ingredients in the feed: soybean meal: crude fat, 1.9%, crude protein, 44.2%; flour: crude fat, 3%, crude protein, 13%; fish meal: crude fat, 8.4%, crude protein, 67%.

2 Mineral premix: CuSO_4_·5H_2_O 20 mg/kg, Na_2_SeO_3_(1%) 50 mg/kg, KI, 100 mg/kg, CoCl_2_ (1%) 100 mg/kg, ZnSO_4_·H_2_O 150 mg/kg, MgSO_4_·H_2_O 4000 mg/kg, MnSO_4_·4H_2_O 50 mg/kg, FeSO_4_·H_2_O 260 mg/kg.

3 Vitamin premix: thiamine 25 mg/kg, ethoxyquin 150 mg/kg, pyridoxine hydrochloride 20 mg/kg, riboflavin 45 mg/kg, vitamin B_12_ 0.1 mg/kg, inositol 800 mg/kg, nicotinic acid 200 mg/kg, vitamin A acetate 32 mg/kg, vitamin K_3_ 10 mg/kg, pantothenic acid 60 mg/kg, biotin 1.2 mg/kg, Vitamin D3 5 mg/kg, α-tocopherol 120 mg/kg, folic acid 20 mg/kg.

Feed raw materials such as FM and SM were put into the pulverizer (SGF130, Tianfan Pharmaceutical Machine Factory, Shanghai, China). In accordance with the principle of mixing from small to large, step by step, the various ingredients in the feed formula were fully mixed; in particular, fish oil and soybean oil were added last; after that, an appropriate amount (about 35% of the weight of the meal) of water with fully dissolved LBP was added mixed and into a paste. The resultant paste was placed in multifunctional spiral extrusion machinery (CD4 × 1 TS, South China University of Technology, Guangzhou, China) to make pellets with a 5-mm diameter (temperature <65 °C; pressure: 0.8 Mpa). The prepared sinking pellet diet was dried in a constant temperature oven at 55 °C, then removed, cooled and dried at room temperature, and frozen at −20 °C.

### 2.4 Experimental fish and feeding management

The culture experiment was carried out in the seawater test field of Jimei University, Xiamen, China. Experimental spotted sea bass were purchased from a commercial nursery farm in Fujian, China. The test period was 52 d. Before the start of the experiment, the test site and tanks were cleaned, disinfected, and aerated. Spotted sea bass were fed in 1200 L temporary tanks for two weeks to adapt to the experimental environment. During the temporary feeding period, fish were fed twice daily (08:30 and 17:30) until satiation. Water was oxygenated throughout the day and changed daily after 30 min of feeding in the afternoon. The salinity of the water was reduced by about 11‰ each time.

After the temporary rearing period, spotted sea bass were fasted for 24 h, then anesthetized with 150 mg/L eugenol, which was purchased from Heshanfu Technology Co., Ltd. (Guangzhou, China) (Holloway et al., 2004). A total of 225 healthy spotted sea bass with the same specifications (initial body weight: 44.52 ± 0.24 g) were randomly divided into 5 groups with 3 replicates per group and randomly assigned to 15 tanks (80 cm × 45 cm × 45 cm).

Feeding management and temporary feeding were roughly the same, and each group was fed corresponding diets. All the breeding tanks were connected, and the temperature of the water body was maintained by the thermal circulation heater at 27 ± 0.2 °C, salinity was maintained between 5.5‰ and 22.0‰, pH was maintained between 7.8 and 8.2, dissolved oxygen was maintained at about 7 mg/L, ammonium-nitrogen  <  0.2 mg/L, and nitrite  <  0.08 mg/L. All values were measured using a DR900 colorimeter and a HQd portable meter (HACH Company, CO, USA).

### 2.5 Sample collection

Spotted sea bass were fasted for 24 h and then anesthetized with 150 mg/L eugenol. Each tank was randomly sampled with 11 fish. Blood samples were collected by the tail vein sampling method, and serum was separated by centrifugation (836 × *g*, 10 min, 4 °C) after 16 h. The serum samples were stored at −80 °C for enzyme activity index detection. When the blood was taken, the fish were put to death humanely. Intestinal tracts were collected, and the surface fat was removed and washed with 0.88% ice saline. Nine intestinal samples were stored in liquid nitrogen for a short time and transferred to the refrigerator at −80 °C. Three intestinal samples were stored for detecting enzyme activity index, and six intestinal samples were used for transcriptome, metabolome, and microbiota analysis. The remaining 2 intestinal samples were fixed with 4% paraformaldehyde for histological analysis.

### 2.6 Analysis of basic composition of diet

The content of crude protein, crude fat, ash, and moisture in feed samples was analyzed by the standard method (AOAC, 2005). The samples were dried in a 105 °C oven to determine the moisture content at constant weight (AOAC method 2001.12). Crude protein (N × 6.25) was determined by the Kjeldahl nitrogen determination method (AOAC method 2001.11). Crude lipid was determined by Soxhlet extraction in ether (AOAC method 920.39). The sample was heated at 550 °C in a Muffle furnace for 8 h to determine the ash content (AOAC method 942.05). Calcium and phosphorus were determined by standards (GB/T 13885-2017 and GB/T 6437-2018) using an inductively coupled plasma emission spectrometer (5110 ICP-OES, Agilent, California, USA). Gross energy was measured by an oxygen bomb calorimeter (6400 calorimeter, Parr Instrument Company).

### 2.7 Growth performance and physical parameter analysis

The relevant parameters of growth performance and physical parameters in the study included the weight gain rate (WGR), specific growth rate (SGR), feed conversion ratio (FCR), feed intake (FI), hepatosomatic index (HSI), condition factor (CF), and survival rate (SR). The calculation formulas were as follows:

$$\text{WGR}\;\left(\%\right)=\left(W_{2}-W_{1}\right)\;/\;W_{1}\times 100\text{;}$$

$$\text{SGR}\;\left(\%/d\right)=\left(\ln\;W_{2}-\ln\;W_{1}\right)\;/\;d\times 100\text{;}$$

$$\text{FCR}=F\;/\;\left(W_{2}\;–\;W_{1}\right)\text{;}$$

FI(g/d)=F/d;

$$\text{HSI}\;\left(\%\right)=W_{H}\;/\;W_{F}\times 100\text{;}$$

$$\text{CF}\;\left(g/{\text{cm}}^{3}\right)=W_{F}\;/\;L^{3}\times 100\text{;}$$

$$\text{SR}\;\left(\%\right)=100\times \left(N_{t}\;/\;N_{0}\right)\text{;}$$

where *W*_2_ is the average final weight (g); *W*_1_ is the average initial weight (g); *d* is the test period in days; *F* is the total FI (g); *W*_*H*_ is liver wet weight (g); *W*_*F*_ is wet weight (g) of test fish; *L* is the body length (cm) of the fish; *N*_*t*_ is the number of survivors; and *N*_0_ is the initial quantity.

### 2.8 Analysis of permeability parameters of intestinal mucosal barrier

In this experiment, the activities of D-lactic acid (D-LA), diamine oxidase (DAO), and albumin (ALB) were measured with commercial kits. D-LA was determined using enzyme-linked immunosorbent assay (ELISA) (Dupre et al., 1977). In short, D-LA antibody was pre-coated in micropores, and the substrate TMB was finally converted to yellow under the action of peroxidase and acid. The color depth was positively correlated with the concentration of D-LA in the sample. Absorbance was measured with a microplate reader (EPHCH2T, BioTek, USA). 1,4-butylenediamine was catalyzed by DAO to produce CH_3_, which further reacted with α-ketoglutaric acid and NADH to produce glutamic acid. The NADH decline rate per minute was measured by a spectrophotometer (UV-1200, MAPADA, China) at 340 nm wavelength, and the DAO activity was calculated (Zhu et al., 2021b); When pH = 4, bromocresol green was changed from yellow to green after binding with albumin, and the color depth was proportional to the ALB concentration. The ALB concentration was calculated by measuring the absorbance at 628 nm wavelength (Doumas et al., 1971). In addition to the D-LA kit purchased from Shanghai Youxuan Biotechnology Co., Ltd., the DAO and ALB kits were provided by Nanjing Jiancheng Biotechnology Co., Ltd.

### 2.9 Intestinal morphological analysis

The intestinal morphological analysis was observed by the method of hematoxylin-eosin staining (Liu et al., 2024). After the samples were fixed in a 4% paraformaldehyde solution for 24 h, they were immersed in a 75% ethanol solution for a full rinse and then immersed in ethanol solutions of different concentrations (75%, 95%, and 100%) successively to dehydrate the samples. Next, the dehydrated samples were immersed in a 1:1 xylene-ethanol mixture for 20 min and a xylene-paraffin mixture for 30 min. After that, the samples were quickly embedded in paraffin at 60 °C. The solidified paraffin samples were sliced with a microtome (RM2016, LEICA, Germany), and the slices were flattened in warm water (about 50 °C). The slides were collected and dried in a constant temperature oven at 55 °C for 8 h and the dried slices were dewaxed in a xylene and ethanol solution, stained with hematoxylin and eosin, and sealed with a glass cover. Three sections were selected in each group. Fiji (Image J-win 64) was used to measure villus height, villus thickness, and muscular thickness, and histological changes of intestinal epithelial cells were evaluated according to the degree of villus changes. A forward white light microscope (Eclipse Ci-L, Nikon, Japan) was used to photograph and analyze these slices, and the slices were observed in various multiples with CaseViewer Ver 2.2 (The Digital Pathology Corp., Hungary).

### 2.10 High-throughput 16S rRNA gene amplification and sequencing

Total genomic DNA was extracted from intestinal samples using the Stool DNA Kit (Tiangen Biotech (Beijing) Co., Ltd.). The hypervariable region V3 to V4 of the bacterial 16S rRNA gene was amplified with primer pairs F: 5′-ACTCCTACGGGAGGCAGCA-3′ and R: 5′-GGACTACHVGGGTWTCTAAT-3'. PCR products were purified with the Omega DNA purification kit (Omega Inc., Norcross, GA, USA) and quantified using Qsep-400 (BiOptic, Inc., New Taipei City, Taiwan, ROC). The amplicon library was paired-end sequenced (2 × 250) on an Illumina Novaseq 6000 (Beijing Biomarker Technologies Co., Ltd., Beijing, China).

### 2.11 Intestinal transcriptome analysis and RT-qPCR

The TRIzol kit was used to isolate total RNA from the intestinal tract of test fish; nucleic acid concentration was detected by Nanodrop 2000, and nucleic acid integrity was detected by Agilent 2100 and LabChip GX. The NEBNext Ultra RNA library was used to prepare (Illumina, NEB, USA), generate sequencing libraries, and add index codes to the attribute sequence of each sample. In short, mRNA was enriched and purified using poly T oligonucleotide attached magnetic beads, and cDNA was synthesized by reverse transcription after fragment treatment. In order to select cDNA fragments that were preferentially 240 bp in length, the library fragments were purified with the AMPure XP system (Beckman Coulter, Beverly, USA). Then 3 μL USER Enzyme (NEB, USA) was used with size-selected, adaptor-ligated cDNA at 37 °C for 15 min, followed by 5 min at 95 °C before PCR. PCR was performed with phusion high-fidelity DNA polymerase, universal PCR primers, and an index (X) primer. Finally, PCR products were purified (AMPure XP system), and library quality was assessed on the Agilent Bioanalyzer 2100 system.

#### 2.11.1 Sequencing

Paired-end reads were generated using Illumina Novaseq 6000 platform (San Diego, USA).

#### 2.11.2 Transcriptome assembly and gene function annotation

Transcriptome assembly was accomplished based on the left and right fastq files as input using Trinity (min_kmer_cov set to 2 by default and all other parameters set to default) (Grabherr et al., 2011). Gene function was annotated based on the following databases: NR (NCBI non-redundant protein sequences), Pfam (Protein family), KOG/COG (Clusters of Orthologous Groups of Proteins), Swiss-Prot (a manually annotated and reviewed protein sequence database), KEGG (Kyoto Encyclopedia of Genes and Genomes), and GO (Gene Ontology).

#### 2.11.3 Quantification of gene expression levels

Gene expression levels were estimated by RNA-Seq by expectation-maximization (RSEM) for each sample (Li and Dewey, 2011).

#### 2.11.4 Differential expression analysis

Differential expression analysis of the two groups was performed using the DESeq R package (1.10.1). DESeq provides statistical routines for determining differential expression in digital gene expression data using a model based on the negative binomial distribution. The resulting *P*-values were adjusted using Benjamini and Hochberg's approach for controlling the false discovery rate. Genes with an adjusted *P*-value <0.05 were assigned as differentially expressed.

#### 2.11.5 RT-qPCR

The RT-qPCR reaction was performed using a commercial kit (Q711-02/03, Nanjing Vazyme Biotech Co., Ltd., China) with the SYBR Green I chimeric fluorescence method. Using β-actin as the internal reference factor, RT-qPCR primers were designed (Table 2). Reaction solution (20 μL) was added to a 96-well plate by mixing 2 μL of template cDNA, 0.4 μL of forward and reverse primer (10 μmol/L), 10 μL of 2 × ChamQ Universal SYBR qPCR Master Mix, and 7.2 μL of RNase-free ddH_2_O. The samples were amplified by fluorescent quantitative PCR (LC480, Roche, Switzerland) and the reaction conditions were: pre-degeneration at 95 °C for 30 s, followed by 40 cycles of 10 s at 95 °C and 30 s at 60 °C. Fluorescence was monitored at the end of each cycle. Finally, the dissolution curves were collected at 95 °C for 15 s, 60 °C for 60 s and 95 °C for 15 s. The 2^−ΔΔCt^ method was adopted to calculate the relative expression levels of each gene among groups.

**Table 2:** Primer information.

Gene	Primer sequence (5′ to 3′)	Annealing temperature, °C
β-Actin	F: AACTGGGATGACATGGAGAAG R: TTGGCTTTGGGGTTCAGG	60
TRINITY_DN10053_c0_g2	F: CCGTCATCTTCAGCGTCTTCA R: GGTCGTCGTCTGCCAAGT	60
TRINITY_DN11098_c0_g1	F: TCCACCGACTACGACAACTAC R: GCTCCTCAATGGTCTCCTCAG	60
TRINITY_DN10352_c0_g1	F: ATCAACTCACTCTCGCCATTCA R: CAGTCGCTCCCATCCCATT	60
TRINITY_DN109_c1_g2	F: CCACTTCACCTGAGTCCTTAACA R: CGTTCGTCCTCTGGCTTCA	60
TRINITY_DN10138_c0_g1	F: AAGGTCTACGCTGTCCATCAA R: TGTATCCACTTGTTCCATCTGAGTT	60

F = forward primer; R = reverse primer.

### 2.12 Intestinal metabolomic analysis

#### 2.12.1 Metabolite extraction

A 50-mg sample was precisely weighed. Subsequently, 1000 μL of extraction solution containing the internal target (methanol to acetonitrile to water volume ratio = 2:2:1, internal standard concentration = 20 mg/L) were added. Following 30 s of vortex mixing, a steel ball was incorporated. The sample was then subjected to a 45 Hz grinder for 10 min. Subsequently, it was immersed in an ultrasonic bath, which was cooled with an ice water mixture, for 10 min. The solution was then stood at −20 °C for 1 h, centrifuged (4 °C, 836 × *g*, 15 min), then 500 μL of the supernatant was extracted into an EP tube, dried in a vacuum concentrator, and 160 μL of the dried metabolites were added into the extraction solution (acetonitrile: water volume ratio = 1:1). The sample was then subject to vortex mixing for 30 s, an ultrasonic bath for 10 min (ice water bath) and centrifugation for 15 min (4 °C, 836 × *g*). Finally, 120 μL of supernatant was put into a sample bottle, and 10 μL of each sample was mixed into the quality control sample for machine testing.

#### 2.12.2 LC-MS analysis

Metabolomic analysis was performed with a Waters Acquity I-Class PLUS ultra-high performance liquid tandem Waters Xevo G2-XS QTOF high resolution mass spectrometer. The Acquity UPLC HSS T3 column was purchased from Waters Co., Ltd., USA. A mass spectrometer to collect primary and secondary mass spectrometry data in MSe mode under the control of the acquisition software (MassLynx V4.2, Waters, USA). In each data collection cycle, simultaneous dual channel data collection occurs, including low collision energy (2 V) and high collision energy (10–40 V). For the mass spectrum, the scanning frequency was 0.2 s. The parameters of the electrospray ionization (ESI) source was as follows: ion source temperature 150 °C; backflush gas flow rate 50 L/h; capillary voltage 2000 (positive ion mode) or −1500 V (negative ion mode); cone voltage 30 V; desolvent gas temperature 500 °C; desolventizing gas flow rate 800 L/h.

#### 2.12.3 Metabolomic data analysis

The MassLynx V4.2 collected raw data was analyzed using Progenesis QI software for data processing. The analysis involved using Biomark's self-built library and Progenesis QI software online METLIN database for identification. The mass deviation and theoretical fragment identification were both within 100 mg/kg. The identified compounds were queried for classification and route information in the KEGG, HMDB, and LipidMaps databases. The significance of the difference between each compound was assessed by comparing the *P*-value using a *t*-test. The R programming language package was utilized to conduct orthogonal partial least squares-discriminant analysis (OPLS-DA) modeling, and the reliability of the model was validated with 200 times permutation tests. The differential metabolites were screened using a combination of the difference multiple, the *P-*value, and the variable importance in projection (VIP) value of the OPLS-DA model. The screening criteria consist of three conditions: log_2_(fold change) > 1, *P*-value <0.05, and VIP >1. The hypergeometric distribution test was used to calculate the significant differential metabolites enriched in the KEGG pathway. All of the above analysis was performed using BMKCloud (http://www.biocloud.net/).

### 2.13 Data statistics and analysis

SPSS Ver 26 was used for all statistical analyses, and 0.05 was deemed the significance level. One-way ANOVA analysis was used to compare differences between groups, and if results showed differences significantly, Duncan's multiple range test was performed. An exception was the use of the students' *t*-test to assess the differences between pairwise pairs in measures related to gut microbial alpha diversity. Figures were made with GraphPad Prism 8. Data were presented as mean values with standard error of the mean (SEM). The effects of different dietary LBP levels were assessed linearly and quadratically using regression analysis.

The linear regression model is as follows:

$$Y=\beta_{0}+\beta_{1}X+\epsilon \text{.}$$

The quadratic regression model is as follows:

$$Y=\beta_{0}+\beta_{1}X+\beta_{2}X^{2}+\epsilon \text{.}$$

where *Y* represents the observation of the dependent variable; *β*_0_, constant/intercept; *X*, independent variable; *β*_1_ and β_2_, regression coefficient; *ϵ*, random error.

## 3 Results

### 3.1 Growth performance and physical parameter analysis

The results of fish growth performance and physical parameters are shown in Table 3. Compared with the LS group, HIS, and CF in the HS group were significantly decreased (*P* < 0.05). After adding LBP to the high SM diet, SGR, FI, and HSI had an upward trend but were not significant (*P* > 0.05). However, CF was significantly decreased (*P* < 0.001). In addition, FCR and WGR were not significantly different among the groups (*P* > 0.05).

**Table 3:** Effects of LBP in high soybean meal diet on growth and physical parameters of spotted sea bass.

Item	Groups^1^					SEM	*P*-value^2^		
	LS	HS	HL1	HL2	HL3		ANOVA	Linear	Quadratic
WGR, %	1.87	1.58	1.67	1.69	1.64	0.499	0.135	0.592	0.738
SGR, %/d	2.02	1.82	1.89	1.90	1.87	0.035	0.128	0.559	0.712
FCR	1.31	1.32	1.32	1.27	1.36	0.029	0.442	0.901	0.849
FI, g/d	29.86	23.05	27.09	26.00	26.05	0.883	0.178	0.249	0.316
HSI, %	1.04^a^	0.76^a^	0.86^a^	0.78^a^	0.81^a^	0.032	0.005	0.588	0.532
CF, g/cm^3^	0.014^a^	0.013^a^	0.012^a^	0.012^a^	0.012^a^	0.0003	<0.001	<0.001	<0.001
SR, %	100	100	100	100	100	–	–	–	–

LBP = *Lycium barbarum* polysaccharides; SEM = standard error of mean; WGR = weight gain rate; SGR = specific growth rate; FCR = feed conversion ratio; FI = feed intake; HSI = hepatosomatic index; CF = condition factor; SR = survival rate.

Different letter superscripts indicate a significant difference (*P* < 0.05).

1 LS: fish meal control group; HS: soybean meal-induced enteritis group induced by high soybean meal diet; HL1: 0.10% *Lycium barbarum* polysaccharides (LBP), supplement group; HL2: 0.15% LBP, supplement group; HL3: 0.20% LBP, supplement group.

2 The *P*-value of ANOVA is the difference among all groups. The *P*-values of linear and quadratic were the difference among soybean meal groups.

### 3.2 Analysis of permeability parameters of the intestinal mucosal barrier

Compared with the LS group, serum D-LA content in the HS group was significantly increased (*P* < 0.001), and serum DAO had an increased trend but were not significant (*P* > 0.05). Furthermore, compared with the HS group, serum D-LA content decreased significantly after LBP supplementation (*P* < 0.001), and serum DAO activity showed a quadratically change (*P* = 0.029) with the lowest value in 0.15% LBP supplementation group. However, the contents of serum and intestinal ALB were not significant changed (*P* > 0.05), when LBP was added (Table 4).

**Table 4:** LBP reduces the permeability of the intestinal mucosa of spotted sea bass.

Item	Groups^1^					SEM	*P*-value^2^		
	LS	HS	HL1	HL2	HL3		ANOVA	Linear	Quadratic
Serum D-LA, μmol/L	23.48^a^	74.33^a^	21.37^a^	22.53^a^	19.92^a^	5.683	<0.001	<0.001	<0.001
Serum DAO, U/L	9.78^a^	15.25^a^	5.59^a^	4.19^a^	17.81^a^	1.828	0.041	0.924	0.029
Serum ALB, g/L	15.16	13.42	13.42	11.38	13.42	0.520	0.269	0.653	0.775
Intestinal ALB, g/L	5.55	2.68	5.77	5.00	6.59	0.546	0.204	0.036	0.113

LBP = *Lycium barbarum* polysaccharides; SEM = standard error of mean; D-LA = D-lactic acid; DAO = diamine oxidase; ALB = albumin.

a-c Different letter superscripts indicate a significant difference (P < 0.05).

1 LS: fish meal control group; HS: soybean meal-induced enteritis group induced by high soybean meal diet; HL1: 0.10% *Lycium barbarum* polysaccharides (LBP), supplement group; HL2: 0.15% LBP, supplement group; HL3: 0.20% LBP, supplement group.

2 The *P*-value of ANOVA is the difference among all groups. The *P*-values of linear and quadratic were the difference among soybean meal groups.

### 3.3 Intestinal morphological analysis

**Fig. 1:**
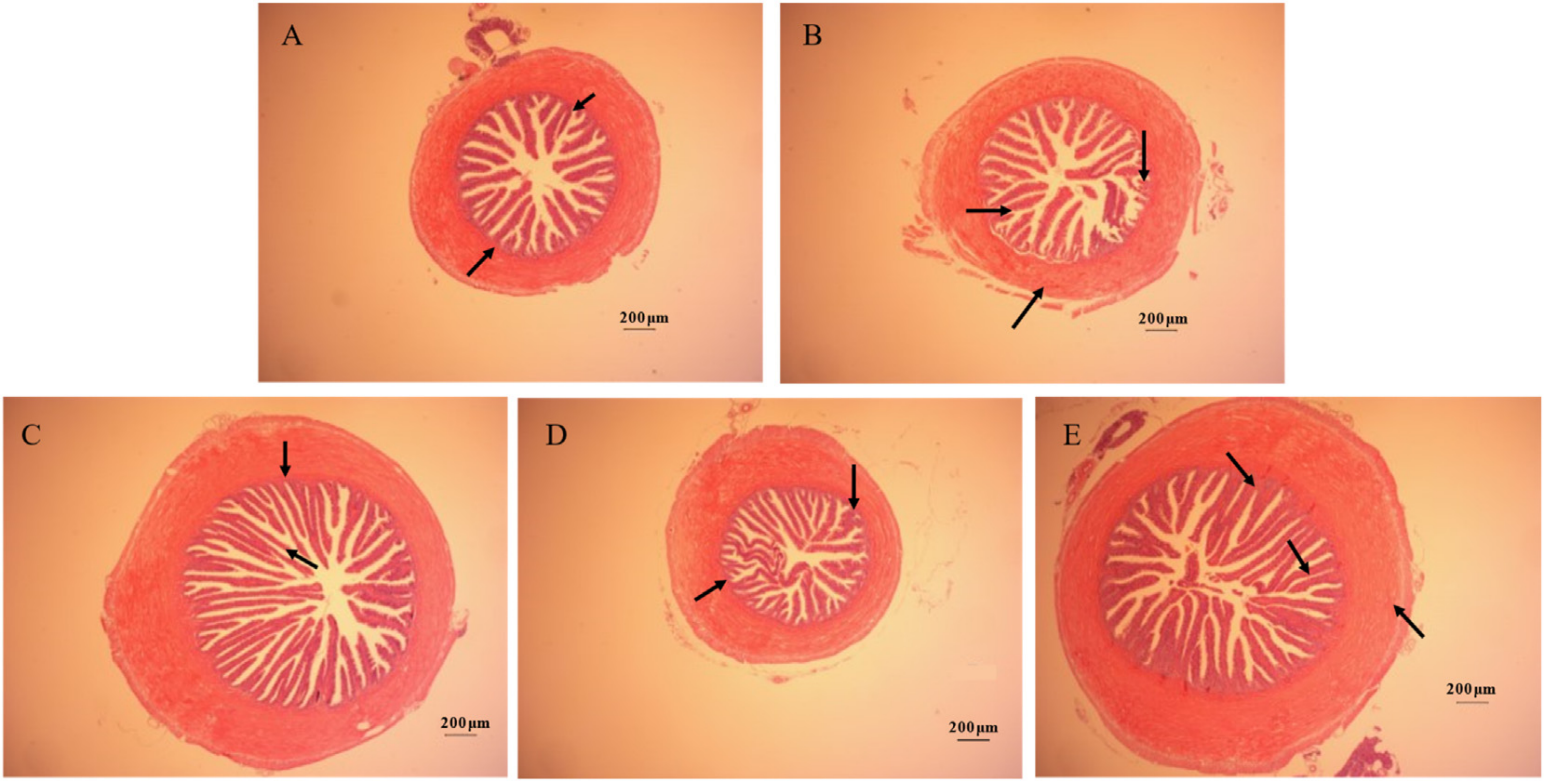
Intestinal morphological of spotted sea bass. The letters in image corners (A to E) represent LS, HS, HL1, HL2, and HL3, respectively. Scale bar = 200 μm. The black arrow indicates intestinal villi shedding and bifurcation. LS: fish meal control group; HS: soybean meal-induced enteritis group induced by high soybean meal diet; HL1: 0.10% *Lycium barbarum* polysaccharides (LBP) supplement group; HL2: 0.15% LBP supplement group; HL3: 0.20% LBP supplement group.

What can be clearly observed is that the mucosal folds of the LS group were intact and the epithelial cells were arranged in an orderly manner. Conversely, in the HS group, the mucosal folds exhibited significant disruption and detachment, appearing short and sparse. The epithelial cells suffered severe damage, with some even detaching. However, the addition of LBP improved the above symptoms to a certain extent; the mucosal folds were relatively complete and closely connected to the muscle layer, no shedding occurred, and the epithelial cells were obviously arranged in a more regular and orderly manner. In addition, compared with the LS group, the intestinal villus height and muscular thickness were significantly reduced in the HS group (*P* < 0.05). After adding 0.10% and 0.20% LBP, intestinal villus height and muscular thickness significantly increased (*P* < 0.05), while intestinal villus thickness decreased significantly (*P* < 0.05) (Table 5 and Fig. 1).

**Table 5:** Intestinal morphological and structure of spotted sea bass.

Item	Groups^1^					SEM	*P*-value^2^		
	LS	HS	HL1	HL2	HL3		ANOVA	Linear	Quadratic
Villus height, μm	449.87^a^	297.73^a^	478.97^a^	250.09^a^	422.60^a^	24.294	<0.001	0.437	0.657
Villus thickness, μm	63.80^a^	61.61^a^	38.81^a^	42.05^a^	50.99^a^	2.908	<0.001	0.065	0.001
Muscular thickness, μm	354.03^a^	234.16^a^	333.63^a^	223.37^a^	320.54^a^	15.051	<0.001	0.214	0.449

SEM = standard error of mean.

a-d Different letter superscripts indicate a significant difference (P < 0.05).

1 LS: fish meal control group; HS: soybean meal-induced enteritis group induced by high soybean meal diet; HL1: 0.10% *Lycium barbarum* polysaccharides (LBP), supplement group; HL2: 0.15% LBP, supplement group; HL3: 0.20% LBP, supplement group.

2 The *P*-value of ANOVA is the difference among all groups. The *P*-values of linear and quadratic were the difference among soybean meal groups.

### 3.4 Intestinal microbiota analysis

#### 3.4.1 Operational taxonomic unit (OTU) analysis

**Fig. 2:**
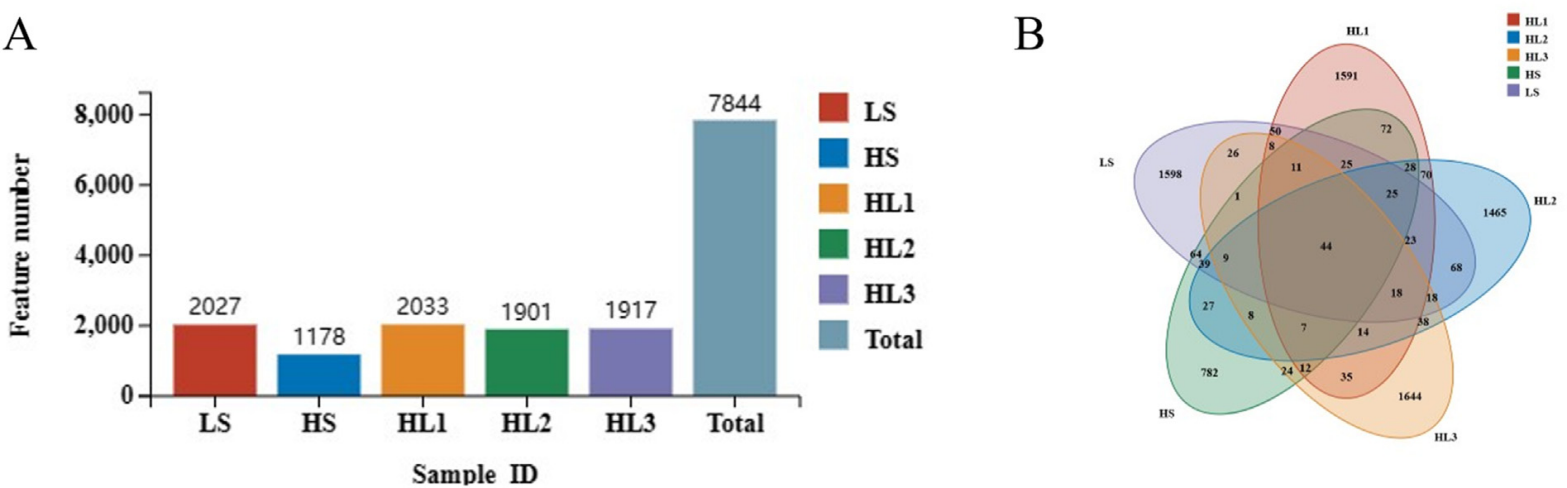
The OTU numbers (A) and Venn diagram (B) of intestinal microbiota in the five groups. OUT = operational taxonomic unit. LS: fish meal control group; HS: soybean meal-induced enteritis group induced by high soybean meal diet; HL1: 0.10% *Lycium barbarum* polysaccharides (LBP) supplement group; HL2: 0.15% LBP supplement group; HL3: 0.20% LBP supplement group.

The OTU analysis indicated that the total number of OTUs in five groups was 7844. Five groups shared 44 OTUs. It is worth mentioning that the total OTU number in the HS group was minimal, and there was little difference in the number of OTUs between the other four groups (Fig. 2).

#### 3.4.2 Microbial species analysis

**Fig. 3:**
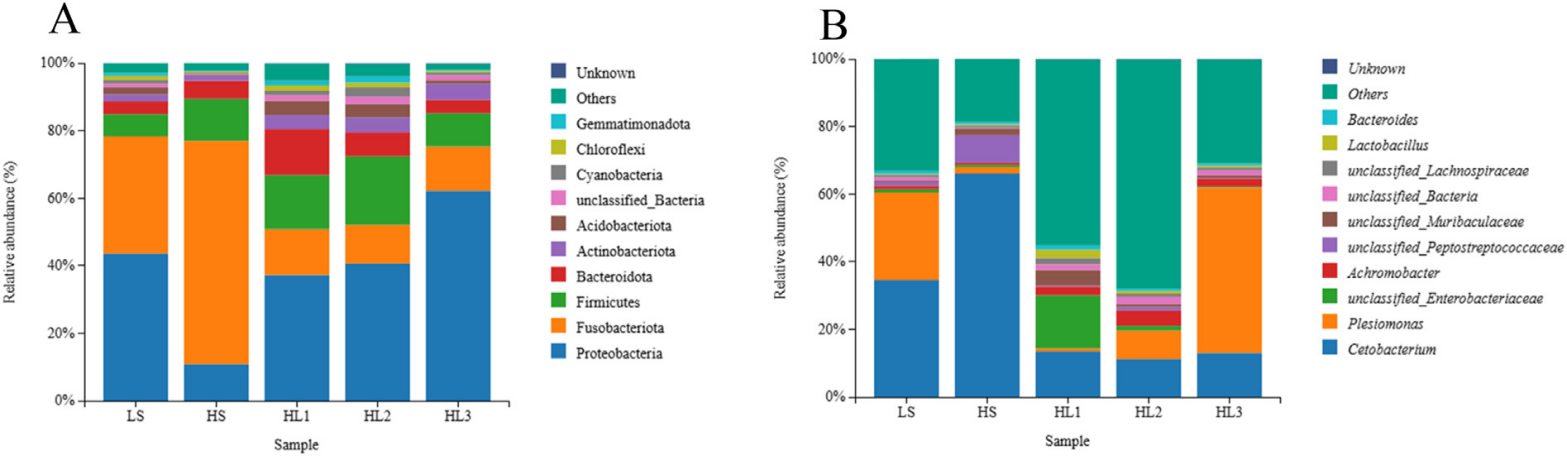
The phylum (A) and genus (B) horizontal species distribution map. Different colors indicate different species; The stacked columns are the taxa with the top 10 relative abundance at each taxa level. LS: fish meal control group; HS: soybean meal-induced enteritis group induced by high soybean meal diet; HL1: 0.10% *Lycium barbarum* polysaccharides (LBP) supplement group; HL2: 0.15% LBP supplement group; HL3: 0.20% LBP supplement group.

Proteobacteria and Fusobacteria were the main intestinal microbiota in the LS group at the phylum level. The proportion of Proteobacteria was reduced in the HS group with high SM content, while the proportion of Fusobacteria was greatly increased. However, after different concentrations of LBP were added, the dominant bacteria group changed to Proteobacteria, while the proportion of Fusobacteria was reduced. At the genus level, *Plesiomonas* and *Cetobacterium* were the main bacteria in the LS group, while *Cetobacterium* was the dominant bacterium in the HS group, and *Plesiomonas* occupied a small proportion. After LBP was added, *Cetobacterium* reduced in proportion, and *Plesiomonas* increased with LBP concentration. It is worth mentioning that when 0.20% LBP was added, *Plesiomonas* accounted for a greater proportion in the HL3 group than the LS group and became the dominant bacterial group (Fig. 3).

#### 3.4.3 Intestinal microbiota diversity analysis

**Fig. 4:**
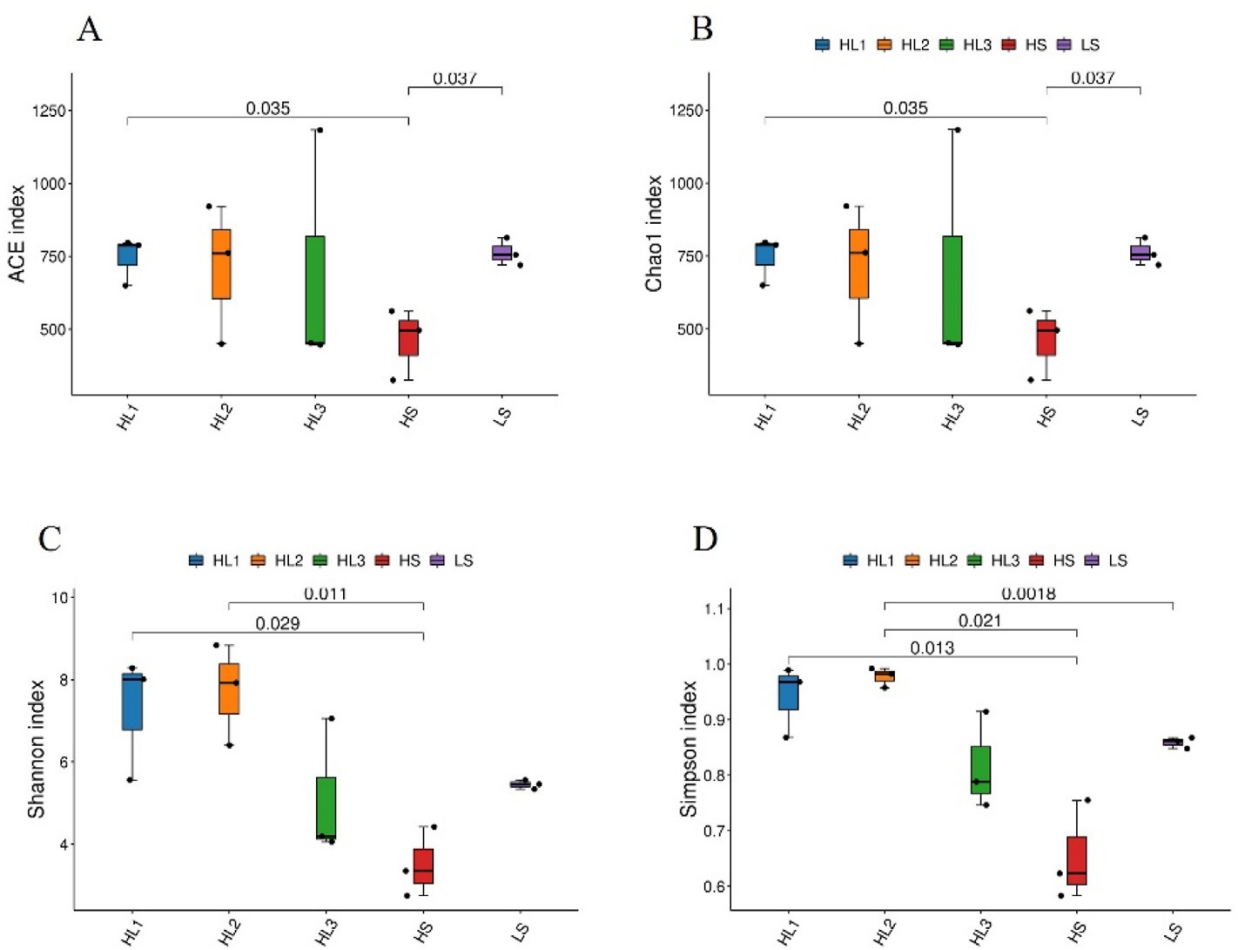
Boxplot of differences between groups of the alpha diversity index. (A) abundance-based coverage estimator (ACE) index; (B) Chao estimator type I (Chao 1) index; (C) Shannon index; (D) Simpson index. The numbers on the lines between the columns are the *P*-values of the *t*-test (if *P* > 0.05, the *P*-values were not displayed). LS: fish meal control group; HS: soybean meal-induced enteritis group induced by high soybean meal diet; HL1: 0.10% *Lycium barbarum* polysaccharides (LBP) supplement group; HL2: 0.15% LBP supplement group; HL3: 0.20% LBP supplement group.

The results of the sample Alpha diversity evaluation showed that, compared with the LS group, the abundance-based coverage estimator (ACE) index and Chao estimator type I (Chao 1) index of the HS group decreased significantly (*P* = 0.037), while the Shannon index and Simpson index showed a decreasing trend. After the addition of LBP, ACE, Chao1, Shannon, and Simpson indexes in the HL1 group, they all increased significantly (*P* = 0.035, 0.035, 0.029, and 0.013, respectively), and Shannon, and Simpson indexes increased significantly in the HL2 group (*P* = 0.011 and 0.021, respectively). Surprisingly, there was no significant change in the HL3 group. The results showed that higher SM content decreased the intestinal species richness of spotted sea bass, but the addition of LBP significantly increased the species richness and species diversity (Fig. 4).

### 3.5 Intestinal transcriptome analysis and RT-qPCR

Based on the results of the above analysis, we selected the LS, HS, and HL1 groups with 3 replicates in each group, and a total of 9 samples were selected for transcriptomic analysis. After sequencing, a total of 61.76 Gb of clean data was obtained, of which 5.79 Gb was clean data and the percentage of Q30 bases was greater than 92.51%. After assembly, 60,442 unigenes were obtained. Of these, 21,506 were longer than 1 kb. For functional annotation of unigenes, 24,684 unigenes annotation results were obtained.

#### 3.5.1 Differentially expressed genes (DEGs)

**Fig. 5:**
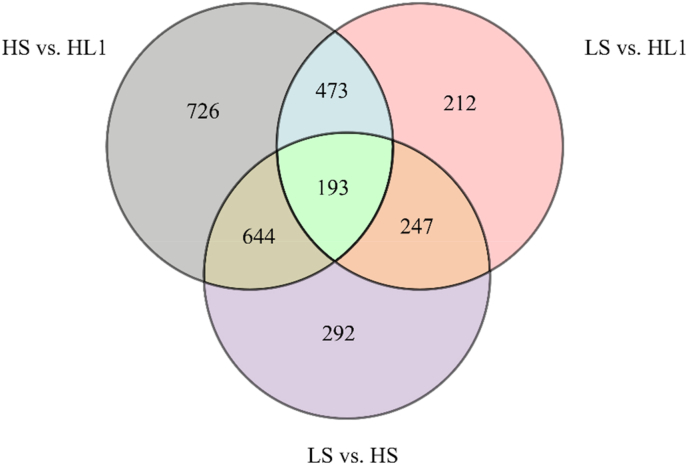
Venn diagram of differentially expressed genes. The numbers on each region represent the number of genes in the corresponding classification, and the overlapping regions represent the number of differential genes shared between related combinations in the region. LS: fish meal control group; HS: soybean meal-induced enteritis group induced by high soybean meal diet; HL1: 0.10% *Lycium barbarum* polysaccharides (LBP) supplement group; HL2: 0.15% LBP supplement group; HL3: 0.20% LBP supplement group.

Compared with the LS group, 1376 DEGs (490 up-regulated, 886 down-regulated) were observed in the HS group. After adding LBP, 2036 DEGs (1194 upregulated, 842 down-regulated) were observed in the HL1 group. In addition, 1,125 DGEs (571 up-regulated, 554 down-regulated) were observed between the LS and HL1 groups. In particular, there were 193 DEGs between the three groups. The distribution of the number of total DEGs is shown in Fig. 5.

#### 3.5.2 GO classification enrichment analysis

**Fig. 6:**
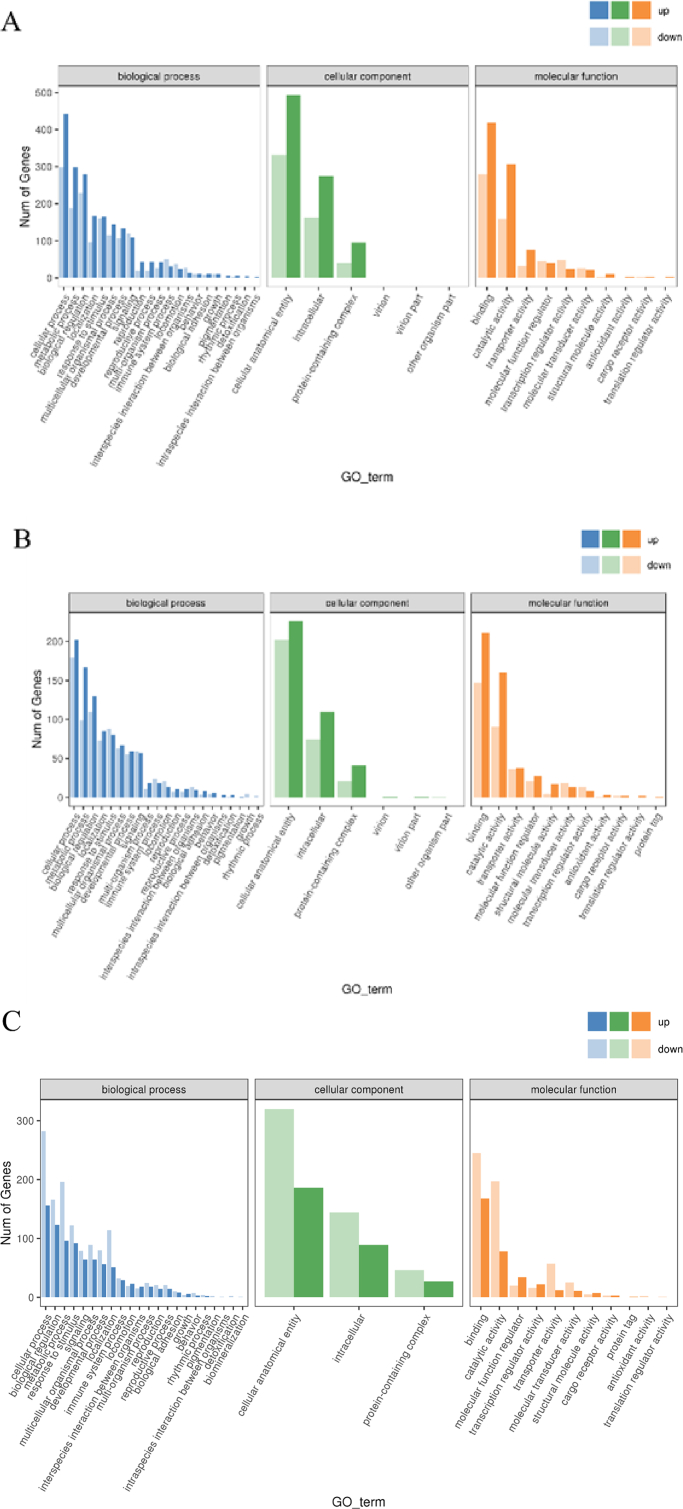
GO annotated classification chart of differentially expressed genes. (A) HS vs. HL1, (B) LS vs. HL1, (C) LS vs. HS. GO = gene ontology. LS: fish meal control group; HS: soybean meal-induced enteritis group induced by high soybean meal diet; HL1: 0.10% *Lycium barbarum* polysaccharides (LBP) supplement group; HL2: 0.15% LBP supplement group; HL3: 0.20% LBP supplement group. The *y*-axis indicates the number of DEGs annotated to one GO term. Genes related to molecular function are shown in blue; genes related to biological processes are shown in green; genes related to cellular components are shown in orange. Down-regulated genes are shown in the light color and up-regulated in the dark color.

The enrichment analysis results of the GO classification showed that, compared with the LS group, the number of down-regulated genes in the HS group was higher than that of up-regulated genes in the three primary classifications: biological processes, molecular function, and cellular component. Cellular process and metabolic process in biological processes, cellular anatomical entity in molecular function, and cellular component binding and catalytic activity in cellular component were observed to have a higher number of DEGs. The up-regulated number of differential genes enriched by these pathways was greater than the down-regulated number. When LBP was added, biological process, molecular function, and cellular component in these pathways showed that the number of up-regulated genes was greater than that of down-regulated genes, and similarly, it was prominent in the same subclasses under the three primary classifications as before. Compared with the LS group, HL1 group also showed that the number of up-regulated genes was greater than the number of down-regulated genes in the above process, and it was also reflected in the same subclass, but overall, the difference between the number of up-regulated genes and down-regulated genes was significantly smaller than that of HS vs. HL1 group (Fig. 6).

#### 3.5.3 KEGG annotation analysis

**Fig. 7:**
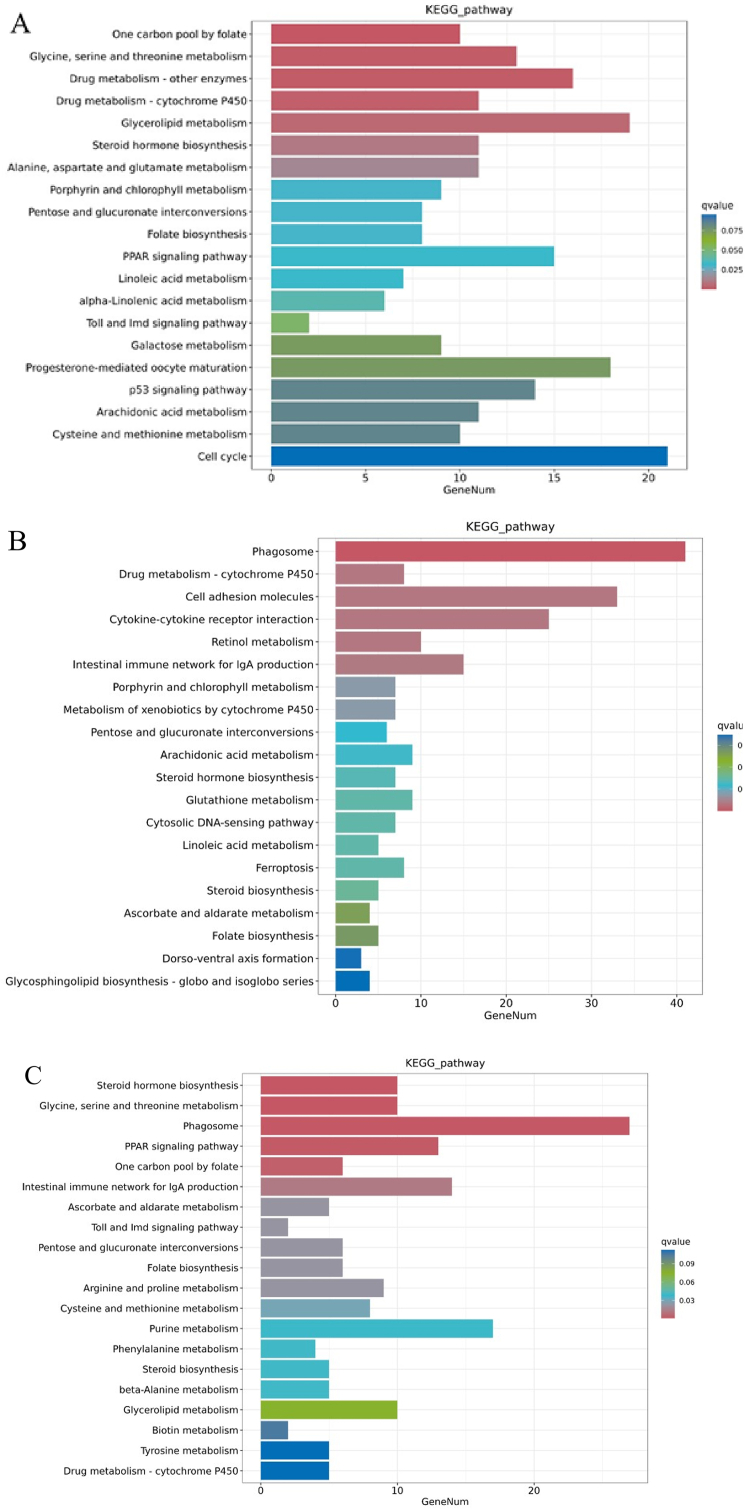
Histogram of KEGG enrichment of differentially expressed genes. (A) HS vs. HL1; (B) LS vs. HL1; (C) LS vs. HS. KEGG = Kyoto encyclopedia of genes and genomes; GeneNum = gene number. LS: fish meal control group; HS: soybean meal-induced enteritis group induced by high soybean meal diet; HL1: 0.10% *Lycium barbarum* polysaccharides (LBP) supplement group; HL2: 0.15% LBP supplement group; HL3: 0.20% LBP supplement group.

The KEGG annotation analysis results showed that the different pathways between the LS group and the HS group mainly included steroid hormone biosynthesis, glycine, serine, and threonine metabolism, phagosome, and the peroxisome proliferator-activated receptor (PPAR) signaling pathway. The different pathways between the HS group and HL1 group mainly included one carbon pool by folate, glycine, serine, and threonine metabolism, drug metabolism-other enzymes, and drug metabolism-cytochrome P450. The LS group and HL1 mainly concentrated on phagosome, drug metabolism-cytochrome P450, cell adhesion molecules and cytokine–cytokine receptor interaction. The addition of SM and LBP had certain effects on genes related to various metabolic pathways in spotted sea bass. In addition, it is worth noting that LBP may act through the drug metabolism-cytochrome P450 pathway and amino acid metabolism-related pathways (Fig. 7).

#### 3.5.4 Differential expression analysis of pro-inflammatory factors and intestinal mucosal barrier-related genes

Compared with the LS group, the expressions of pro-inflammatory factors such as interleukin-1β (*IL-1β*), interleukin-22 (*IL-22*), and interleukin-26 (*IL-26*) were significantly up-regulated in the HS group (*P* < 0.05), especially the expression of pro-inflammatory factors *IL-17C* and *IL-17F*, which was significantly up-regulated (*P* < 0.05), but interestingly, the expression of the *IL-17E* receptor gene was significantly down-regulated (*P* < 0.05). However, only *IL-17C* gene expression was down-regulated in the HL1 group (*P* < 0.05), and other pro-inflammatory genes were not significantly different (*P* ＞0.05). Compared with the HS group, the expression of proinflammatory factors such as *IL-1β*, *IL-12*, *IL-22*, and *IL-26* in the HL1 group supplemented with LBP was significantly down-regulated (*P* < 0.05). Nuclear factor kappa B subunit 2 (*NF-κB2*) and dual specificity MAP kinase in MAPK signaling pathway phosphatase (*MAP*) gene expression were down-regulated, as was the expression of Toll-like receptor 2 (*TLR2*) and inhibitor of nuclear factor kappa-B kinase subunit epsilon (*IKKE*) (*P* < 0.05). In addition, compared with the LS and HS groups, *Occludin*, an important tight junction protein gene, was significantly up-regulated in the HL1 group (*P* < 0.05), indicating that LBP may reduce intestinal mucosal permeability by up-regulating the *Occludin* gene.

#### 3.5.5 RT-qPCR verification

**Fig. 8:**
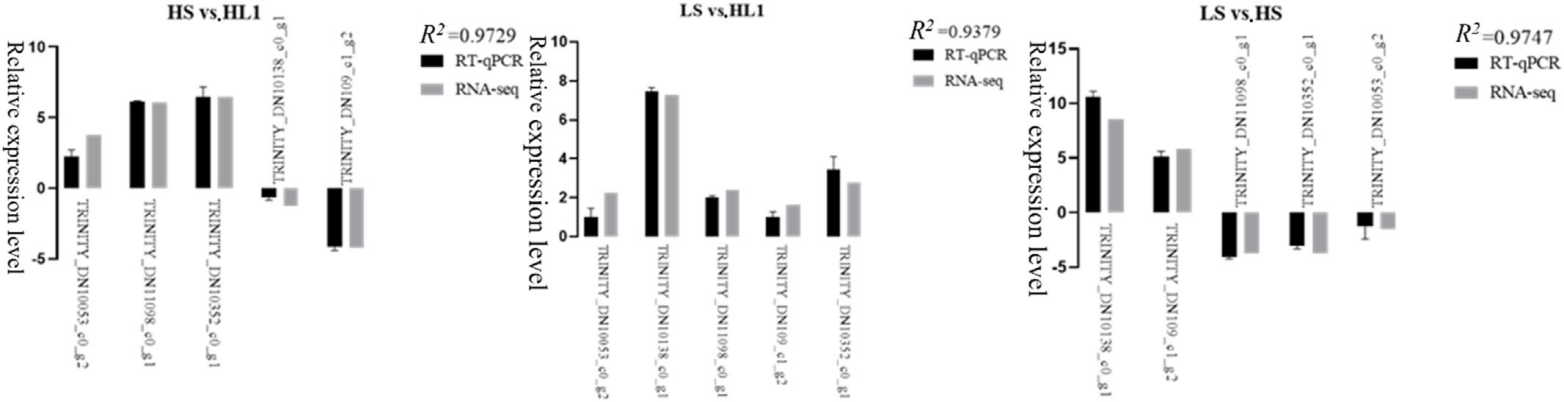
Relative expression levels (log 2 fold change). LS: fish meal control group; HS: soybean meal-induced enteritis group induced by high soybean meal diet; HL1: 0.10% *Lycium barbarum* polysaccharides (LBP) supplement group; HL2: 0.15% LBP supplement group; HL3: 0.20% LBP supplement group.

After RNA-seq transcriptomic analysis of the data, in order to verify the accuracy of the transcriptomic results, RT-qPCR (biological repeat *n* = 6) was used for validation analysis. Five DEGs were randomly selected and matched between the three groups for comparison. The results confirmed that the expression differences of the five DEGs among the three groups were consistent with the RNA-Seq trend, where LS vs. HS, *R*^2^ = 0.9747; HS vs. HL1, *R*^2^ = 0.9729; and LS vs. HL1, *R*^2^ = 0.9379. The results further confirm the reliability and authenticity of the RNA-Seq transcriptome analysis results (Fig. 8).

### 3.6 Intestinal metabolomic analysis

Similarly, the LS, HS, and HL1 groups were selected for metabolomic analysis, and a total of 18,501 peaks were detected, of which 3322 metabolites were noted.

**Fig. 9:**
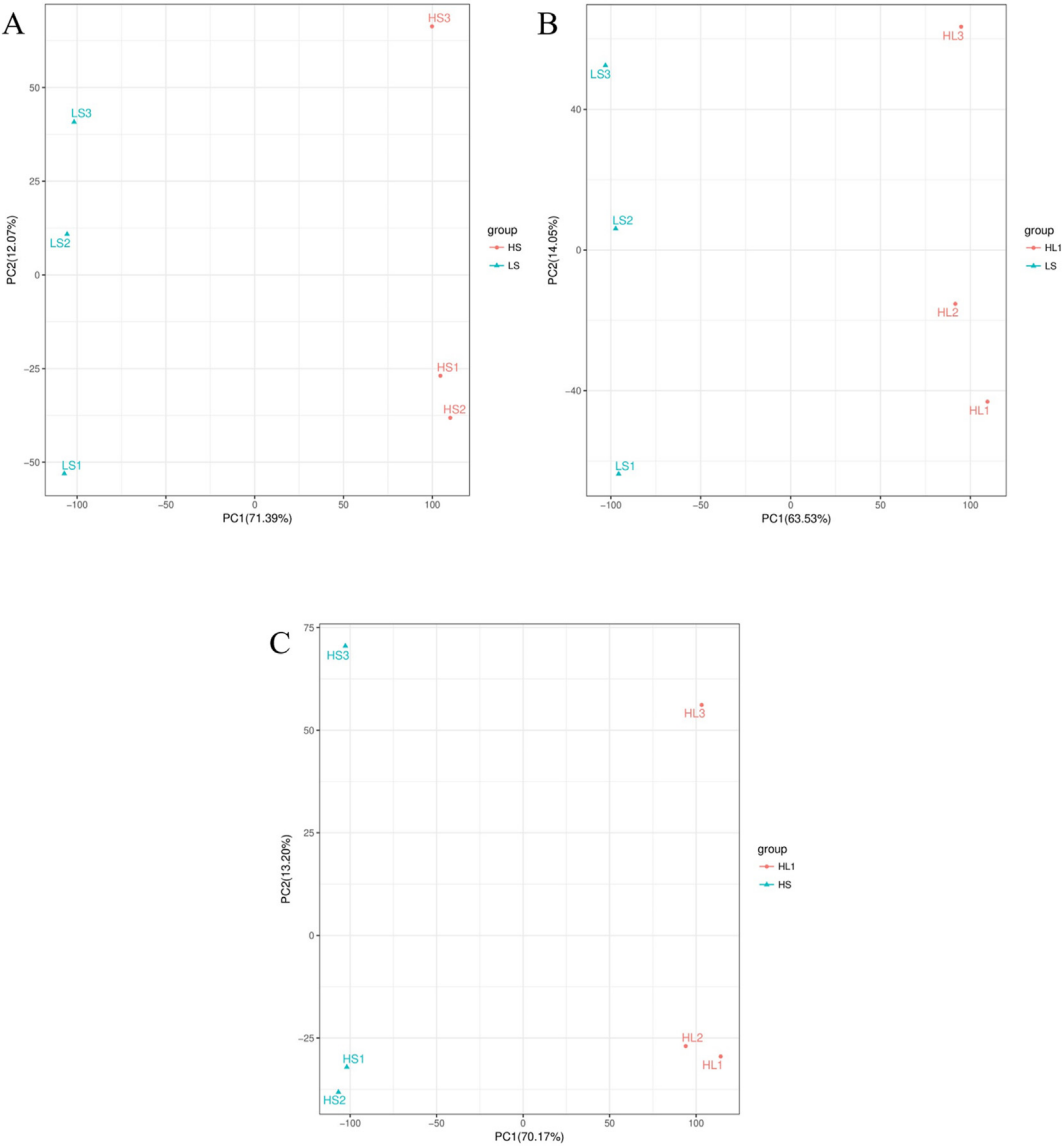
The PCA analysis of pairwise comparisons. (A) LS vs. HS, (B) LS vs. HL1, (C) HS vs. HL1. LS: fish meal control group; HS: soybean meal-induced enteritis group induced by high soybean meal diet; HL1: 0.10% *Lycium barbarum* polysaccharides (LBP) supplement group; HL2: 0.15% LBP supplement group; HL3: 0.20% LBP supplement group.

**Fig. 10:**
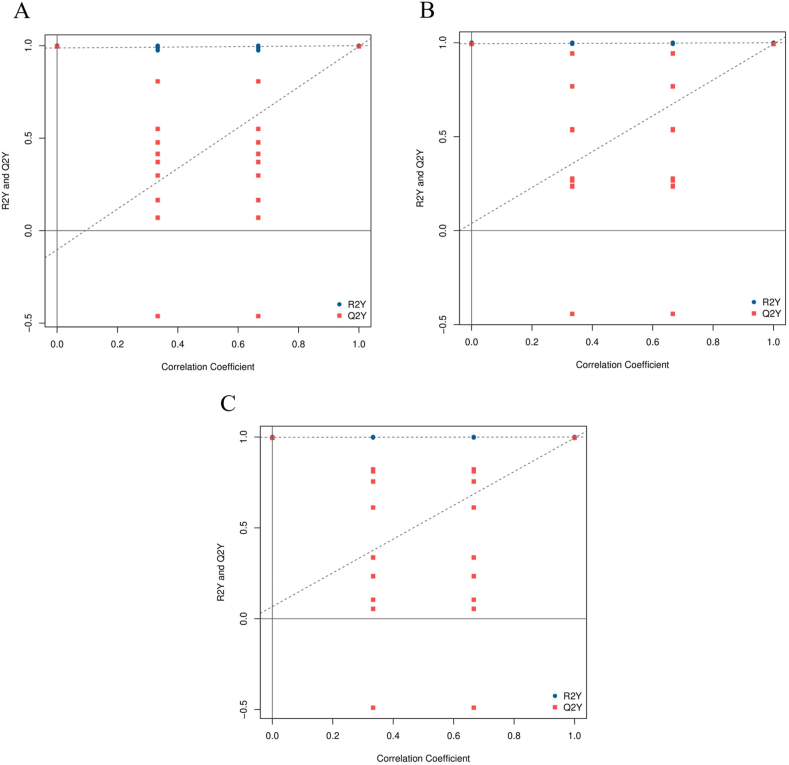
OPLS-DA analysis of pairwise comparisons. (A) HS vs. HL1; (B) LS vs. HL1; (C) LS vs. HS. R2Y represents how well the model explains the variability of the dependent variable (Y). It ranges from 0 to 1, with a value closer to 1 indicating a better fit of the model to the data. Q2Y represents the predictive power of the model, also known as the predictive power of cross-validation. It also ranges from 0 to 1, with a value closer to 1 indicating better predictive performance of the model. LS: fish meal control group; HS: soybean meal-induced enteritis group induced by high soybean meal diet; HL1: 0.10% *Lycium barbarum* polysaccharides (LBP) supplement group; HL2: 0.15% LBP supplement group; HL3: 0.20% LBP supplement group.

**Fig. 11:**
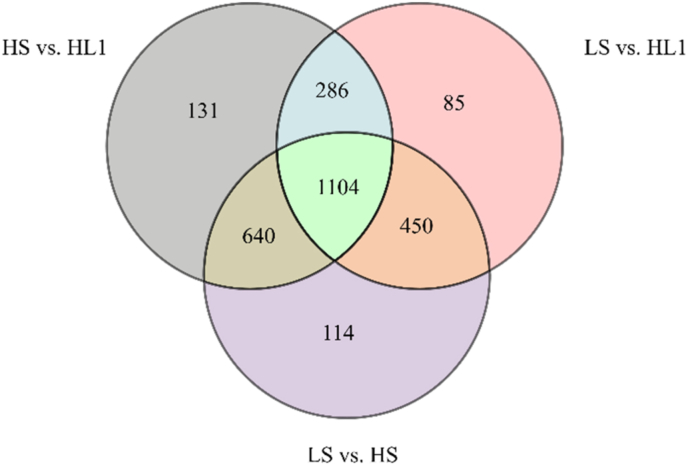
Venn diagram of differential metabolites. The numbers on each region represent the number of genes in the corresponding classification, and the overlapping regions represent the number of differential genes shared between related combinations in the region. LS: fish meal control group; HS: soybean meal-induced enteritis group induced by high soybean meal diet; HL1: 0.10% *Lycium barbarum* polysaccharides (LBP) supplement group; HL2: 0.15% LBP supplement group; HL3: 0.20% LBP supplement group.

The principal component analysis (PCA) analysis results showed that there was no overlap between LS and HS, LS and HL1, HS and HL1 (Fig. 9). The results of （orthogonal partial least squares-discriminant analysis (OPLS-DA) showed that all the Q2Y (parameters of model predictive power) values were over 0.9, providing evidence that the model built using this experimental sample is of good quality (Fig. 10). The results of differential metabolite analysis showed that, compared with the LS group, a total of 2308 differential metabolites were found in the HS group (1066 up-regulated, 1242 down-regulated), and 1925 differential metabolites were found in the HL1 group (953 up-regulated, 972 down-regulated). In addition, a total of 2161 differential metabolites (1239 up-regulated and 922 down-regulated) were found in the HL1 group compared to the HS group. In particular, there were 1104 differential metabolites between the three groups (Fig. 11).

**Fig. 12:**
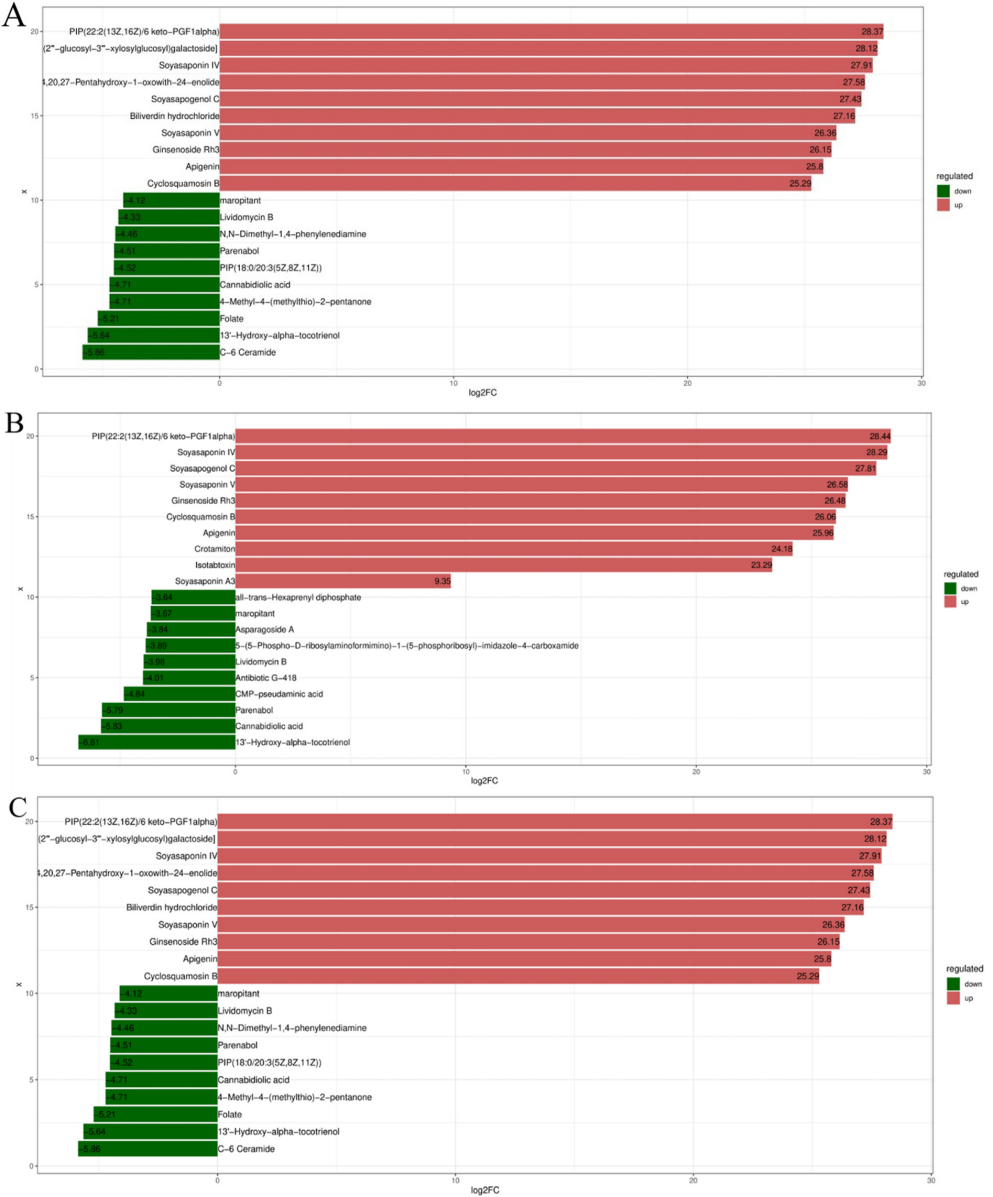
The top ten up- and down-regulated differential metabolites. (A) HS vs. HL1; (B) LS vs. HL1; (C) LS vs. HS. KEGG = Kyoto encyclopedia of genes and genomes; GeneNum: gene number. LS: fish meal control group; HS: soybean meal-induced enteritis group induced by high soybean meal diet; HL1: 0.10% *Lycium barbarum* polysaccharides (LBP) supplement group; HL2: 0.15% LBP supplement group; HL3: 0.20% LBP supplement group.

After the differential log conversion treatment for the fold change (FC) of different metabolites in each group comparison, the top 10 metabolites log_2_ (FC) of each group comparison were screened. The results were as follows: compared with the LS group, with the increase in SM content in the HS group, metabolites including soyasaponin IV, soyasapogenol C, mabioside A, and other saponins were upregulated accordingly. Furthermore, it is worth mentioning that biliverdin hydrochloride was also upregulated, and its secretion can upregulate the activity of biliverdin reductase (BVR). Studies have shown that BVR is a key regulator of acute injury and injury-induced innate immune responses (Wegiel and Otterbein, 2012). Metabolites C-6 ceramide, n-docosahexaenoyl histidine, amphibine H, L-rhamnose, and folate were down-regulated. Interestingly, compared with the HS group, the metabolites of amphibine H and L-rhamnose were up-regulated in the HL1 group supplemented with LBP. Neoisoliquiritin, phosphatidylinositol phosphate (PIP) (18:0/6 keto-PGF1alpha), and lysophosphatidic acid (LysoPA) (0:0/18:1(9Z)) also showed upregulated results. The corresponding down-regulated metabolites included cinncassiol D4 2-glucoside, emorfazone, and taurodehydrocholic acid. Compared with the LS group, the HL1 group showed up-regulation of saponin metabolites including soyasaponin IV, soyasaponin V, soyasapogenol C, mabioside A, neoisoliquiritin, and PIP (18:0/6 keto-PGF1 alpha). The down-regulated metabolites included n-docosahexaenoyl histidine and cinncassiol D4 2-glucoside (Fig. 12).

**Fig. 13:**
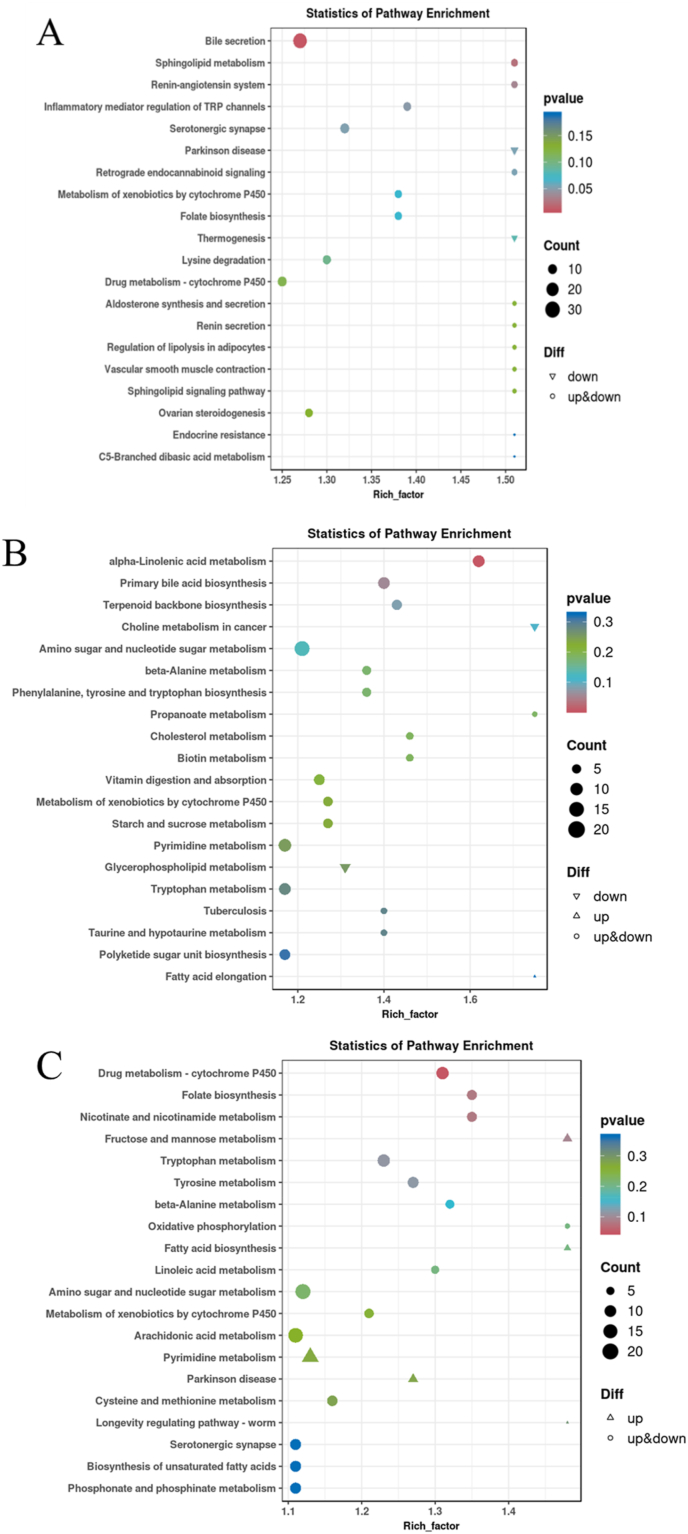
Differential metabolite KEGG enrichment bubble plot. (A) HS vs. HL1; (B) LS vs. HL1; (C) LS vs. HS3. KEGG = Kyoto encyclopedia of genes and genomes; GeneNum: gene number. LS: fish meal control group; HS: soybean meal-induced enteritis group induced by high soybean meal diet; HL1: 0.10% *Lycium barbarum* polysaccharides (LBP) supplement group; HL2: 0.15% LBP supplement group; HL3: 0.20% LBP supplement group.

Enrichment results of the KEGG pathway analysis showed that the differences between LS and HS metabolites were mainly around bile secretion, sphingolipid metabolism, serotonergic synapse and secretion, and inflammatory mediator regulation of the transient receptor potential (TRP) channel. The metabolites that differ between HS and HL1 were mainly drug metabolism-cytochrome P450, folate biosynthesis, nicotinate, and nicotinamide metabolism. The differential metabolites between LS and HL1 were concentrated in alpha-linolenic acid metabolism, primary bile acid biosynthesis, and terpenoid backbone biosynthesis (Fig. 13).

### 3.7 Combined analysis of metabolome and transcriptome

**Fig. 14:**
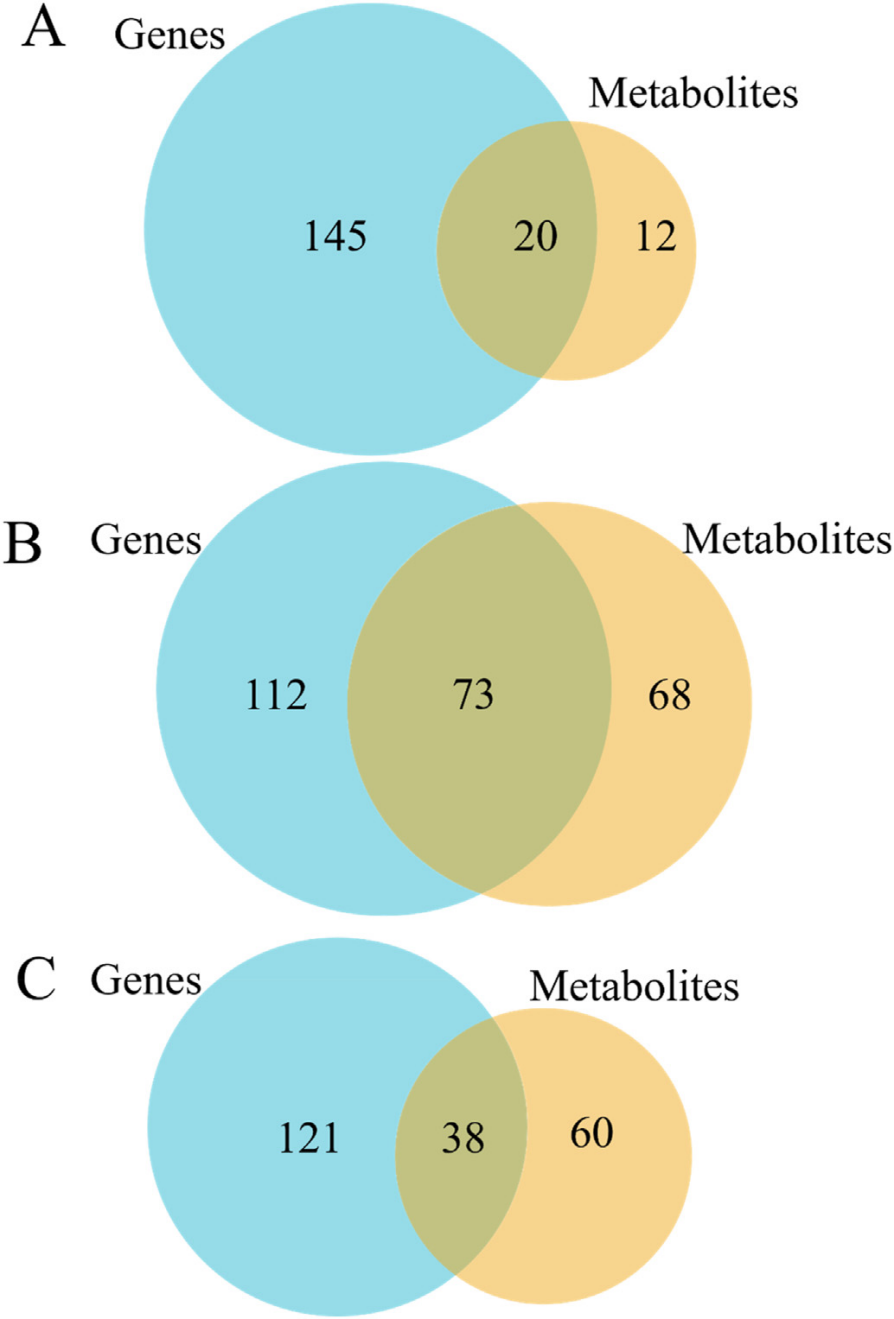
Venn diagram of pathways shared between differential genes and differential metabolites. (A) LS vs. HS; (B) HS vs. HL1; (C) LS vs. HL1. LS: fish meal control group; HS: soybean meal-induced enteritis group induced by high soybean meal diet; HL1: 0.10% *Lycium barbarum* polysaccharides (LBP) supplement group; HL2: 0.15% LBP supplement group; HL3: 0.20% LBP supplement group.

By comparing gene participation pathways in the transcriptome and metabolite participation pathways in the metabolome, the number of co-participating pathways was obtained, and the results showed that LS vs. HS shared 20 pathways, HS vs. HL1 shared 73 pathways, and LS vs. HL1 shared 38 pathways (Fig. 14).

**Fig. 15:**
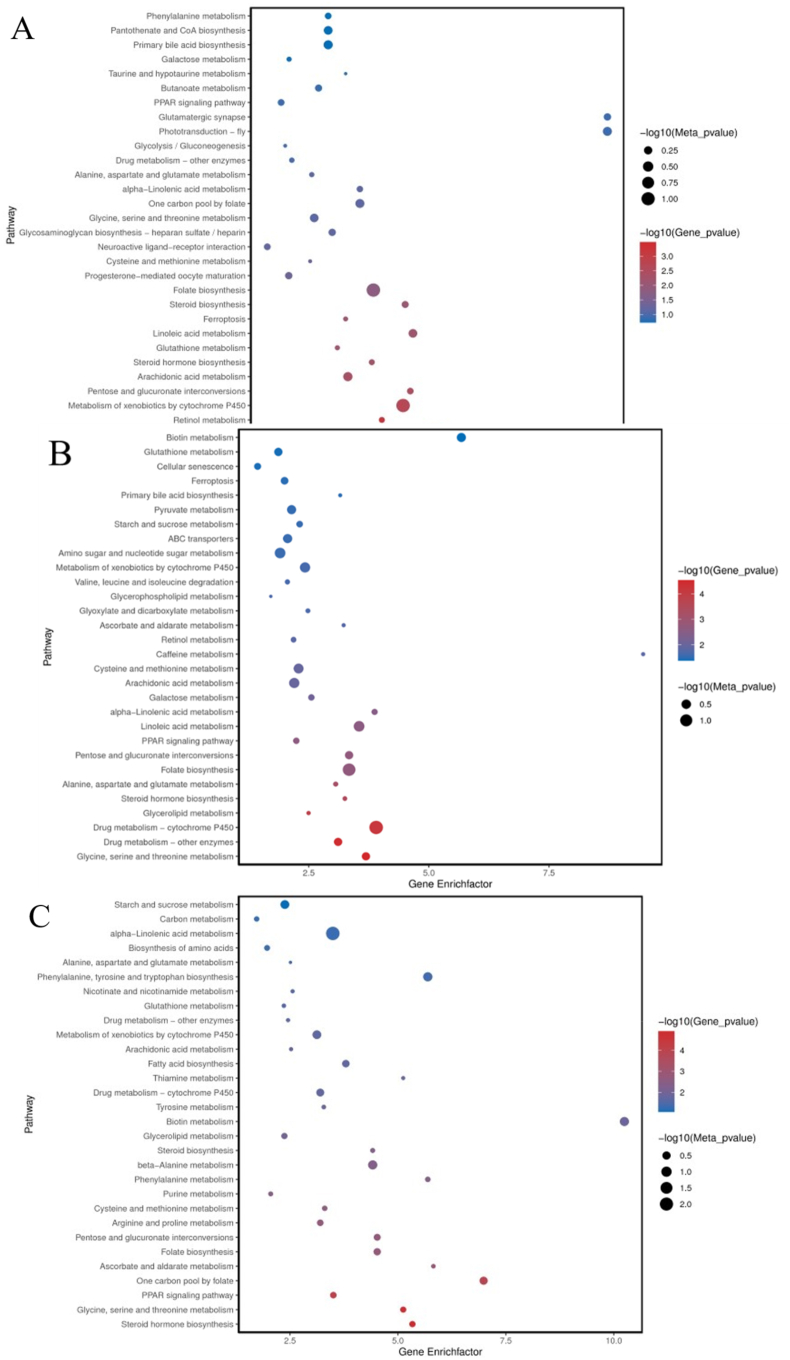
Bubble map of the first 30 KEGG pathways with significant enrichment of differential genes/metabolites. (A) LS vs. HS; (B) HS vs. HL1; (C) LS vs. HL1. The size of the dots represents the enrichment significance of annotated differential metabolites in this pathway. The larger the dots, the more enriched they are. The color of the dots represents the enrichment significance of annotated differential genes in this pathway, and the redder the dots, the more enriched they are. LS: fish meal control group; HS: soybean meal-induced enteritis group induced by high soybean meal diet; HL1: 0.10% *Lycium barbarum* polysaccharides (LBP) supplement group; HL2: 0.15% LBP supplement group; HL3: 0.20% LBP supplement group.

We screened maps of the top 30 pathways with the most significant enrichment of differential genes/metabolites (*P* < 0.05) (Fig. 15). Compared with the LS group, pathways that were significantly enriched in both differential genes and metabolites in the HS group included drug metabolism-cytochrome P450, metabolism of xenobiotics by cytochrome P450, arachidonic acid metabolism, and folate biosynthesis, mainly related to metabolism (*P* < 0.05). The HL1 group mainly focused on steroid hormone biosynthesis, glycine, serine, and threonine metabolism, the PPAR signaling pathway, and one carbon pool by folate (*P* < 0.05). In addition, the most notable enrichment pathways between the HL1 and HS groups with LBP addition included glycine, serine, and threonine metabolism (*P* < 0.05).

## 4 Discussion

Several studies have reported on the use of SM as a substitute for fish meal in the feed of spotted sea bass. Li et al. (2012) evaluated commercial SM and high-value SM as substitutes for FM, and the results showed that commercial SM could replace 30% FM, while high-value SM could successfully replace 45% FM. The results of Zhang et al. (2016) showed that SM could replace 60% of FM in Japanese seabass (*Lateolabrax japonicus*) feed. Zhang et al. (2018) reported that replacing 50% fish meal with SM did not significantly reduce growth performance. Similarly, in this study, growth performance did not show significant changes. Although the reported fish species may be different, the results suggest that to a certain extent, replacing FM with SM has little effect on the growth performance of cultured fish. Interestingly, this study found no significant difference in growth performance following the addition of LBP. On the contrary, several studies of dietary supplementation with LBP have reported significant improvement in the growth performance of cultured fish (Zhang et al., 2020; Huang et al., 2023b). Based on the above reports and the results of this study, two possible explanations were obtained. First, the initial body weight of fish in this study was higher than other reports, and the addition of LBP may not have been enough to promote the growth of cultured fish. The second is that high SM has a negative impact on cultured fish, including SBMIE, and the addition of LBP may preferentially alleviate this negative impact rather than promote their growth, which has been demonstrated in the results of this study.

The intestinal mucosa is a permeable barrier allowing nutrients to enter and exit the intestine while maintaining a barrier effect towards harmful substances. However, if its permeability is too high, external antigens and toxic factors will enter the host body and induce adverse effects such as inflammation (Fiocchi, 1998; Liu et al., 2019). The occurrence of inflammation is the primary response to intestinal intolerance to dietary components (Kiron et al., 2020). DAO is a highly active intracellular enzyme secreted by villus cells in the upper layer of the intestinal mucosa. Its activity is closely related to nucleic acid and protein synthesis in mucosal cells. D-lactic acid is a metabolic product of bacterial fermentation and can be produced by the intestinal flora of animals. In the case of the normal mechanical barrier of the intestinal mucosa, DAO and D-LA can rarely be absorbed into the blood of animals. Therefore, the higher the content of DAO and D-LA in serum, the higher the permeability of the intestinal mucosa. ALB is synthesized by liver parenchymal cells and is the most abundant protein in plasma. When its content increases, it can reduce damage caused by the inflammatory factor interleukin. This study designed a diet containing 40% SM to induce SBMIE. The results of the induction evaluation showed that serum D-LA content was significantly increased, and DAO was slightly increased, indicating higher intestinal mucosal permeability. A large number of intestinal mucosal folds were damaged and shed, the diversity of intestinal flora decreased, and the expression of pro-inflammatory factors *IL-1β*, *IL-2*, *IL-12*, and other genes increased significantly. These changes have been reported in previous studies (Zhang et al., 2018, 2023). In addition, in this experiment, the up-regulation of Soyasaponin IV and Soyasapogenol C in the SM induction group clearly indicated that the intestinal tract or intestinal attached microorganisms could not properly decompose soybean saponins and other ANFs contained in SM, thus inducing enteritis and impacting intestinal barrier function. Therefore, the addition of 40% SM to the diet can induce SBMIE, which was apparently reflected in the damage to the intestinal mucosal barrier and the changes in tissue morphology. These changes are consistent with the symptoms of intestinal inflammation caused by high SM reported by Zhang et al. (2018). According to our experimental results, the addition of LBP could alleviate this situation. Firstly, the decrease in serum D-LA content and DAO activity indicated improvements in the intestinal mucosal barrier and that intestinal mucosal permeability was reduced. Secondly, the observation of intestinal tissue morphology showed that the mucosal fold was relatively complete and closely connected to the muscular layer, and no shedding occurred. Therefore, this study concludes that LBP can alleviate inflammation by repairing damage to the intestinal mucosal barrier. There are many reports that LBP can reduce intestinal mucosal permeability and improve the intestinal mucosal barrier (Li et al., 2020; Zhou et al., 2022, 2023; Gao et al., 2021). Li et al. (2023) reported that LBP promoted the expression of tight junction proteins via nuclear factor erythroid 2-related factor 2 (*Nrf 2*) activation and decreased *Claudin-2* expression to maintain the intestinal mucosal barrier; Zhao et al. (2024) reported arabinogalactan (LBP-3) from *L. barbarum* improved intestinal barrier function; and Kang et al. (2017) reported LBP ameliorated colonic edema, mucosal damage, and neutrophil infiltration.

During the early development stages in fish, the setting process of the intestinal microbiota is complex and may depend on eggs, water environment, and feed (Bergh et al., 1994; Ringo and Birkbeck, 1999). Before the digestive tract is fully developed, fish rely on their gills and mouths to take in food from water, during which time a relatively stable microbiome is established, but the main components come from the aquatic environment (Romero and Navarrete, 2006). In addition, and most importantly, at the beginning of feeding, the diet can greatly alter the intestinal microbiome and even determine the intestinal microbiome structure or composition (Grisez et al., 1997). In past reports, it has apparently been shown that diets containing large amounts of soy meal produce drastic changes in fish intestinal microbes (Liu et al., 2020; Ringo et al., 2016). Similarly, in this experiment, the high SM diet decreased the proportion of intestinal microbes Proteobacteria and increased the proportion of Fusobacteria, and the index of alpha diversity was significantly reduced. Orso et al. (2021) reported that intestinal inflammation in adult zebrafish (*Danio rerio*) also reduced the abundance of Proteobacteria and increased the level of anaerobic bacteria, including *Clostridium*. In addition, relevant studies have reported that the decrease in Firmicutes and Bacteroides in inflammatory zebrafish has been replaced by a significant increase in Fusobacteria (van Kessel et al., 2011). Qiao et al. (2019) also reported a significant increase in *Clostridium* in the inflammatory intestinal model. The composition of the intestinal microbiota varies greatly between different species and individuals of fish, but several microorganisms have been identified as dominant, including Proteobacteria, Firmicutes, Bacteroidetes, Actinobacteria, and Fusobacteria (Eichmiller et al., 2016). Among them, Proteobacteria has been described as the most abundant phylum in fish microbiota characterization studies, and it usually dominates the intestines of marine non-herbivorous fish (Egerton et al., 2018; Piazzon et al., 2019). The fish microbiota that feed on FM were thought to be the closest to their native form, while the distal intestinal microbiota were more actively involved in host digestion when soy meal was introduced into the diet. However, when the content of SM was too high, it was observed that the reduction or disappearance of intestinal primitive microorganisms, such as *Halomonas* spp. and *Pseudomonas* spp., caused intestinal diseases (Kononova et al., 2019). These results indicate that the addition of SM to the diet can take the intestinal microflora away from their original form and produce corresponding changes to adapt to the addition of SM. In this study, the dominant microflora of the intestinal microflora of the spotted sea bass completely fed with fish meal is Proteobacteria. The addition of LBP can restore the intestinal microflora composition of spotted sea bass fed with high SM content to a level close to that of spotted sea bass exclusively fed fish meal, especially the dominant microbial population. In addition, the indices of alpha diversity increased, and the microbial richness and diversity were even greater than those of fish completely fed fish meal. It has been proven that LBP can increase the abundance of Proteobacteria, increase the diversity of intestinal microbes, and even promote the production of SCFAs, thereby maintaining the balance of the intestinal microbiota and intestinal health (Zhu et al., 2020a, Zhu et al., 2020b; Yang et al., 2021; Zhou et al., 2023). This study also confirms this result by demonstrating that LBP mitigated SBMIE by modulating intestinal microbial composition and diversity. Sun et al. (2023) added LBP to the diet of Bulatmai barbel (*Luciobarbus capito*) and found that LBP increased the abundance of Proteobacteria, but the microbial diversity and intestinal tissue morphology did not change significantly, which slightly differs from the results of this study. In this study, spotted sea bass is a typical carnivorous fish, so the dominant microflora was Proteobacteria, whereas Bulatmai barbel is an omnivorous fish, in which the dominant microflora are typically Proteobacteria and Firmicutes, and the intestinal microbial composition and diversity of the two were also different. The polysaccharide chain of LBP is mainly digested and utilized by intestinal microorganisms (Cao et al., 2022). Therefore, differences in composition of intestinal microorganisms will affect the digestion and utilization of LBP and subsequently affect its impact in the intestine.

As mentioned in the introduction, LBP is a kind of prebiotic polysaccharide, and its effects depend on its interaction with intestinal microorganisms: (1) polysaccharides regulate the composition of intestinal microorganisms; (2) intestinal microorganisms metabolize polysaccharides into SCFAs; (3) polysaccharides regulate the intestinal microbiota and their metabolites, such as tryptophan, trimethylamine, and lipopolysaccharides (Ge et al., 2021; Zhang et al., 2022). In addition, natural polysaccharides themselves are also considered to be a class of beneficial nutrients that affect intestinal mucosal barrier function by promoting the development and maturation of epithelial and mucosal epithelial cells (Medrano et al., 2011; Zuo et al., 2015). The findings of the effects of LBP on alleviating inflammation and regulating intestinal microorganisms have been widely reported (Sun et al., 2023; Zhou et al., 2022; Zhu et al., 2020a, Zhu et al., 2020b; Cui et al., 2020). LBP alleviates SBMIE through many ways and interactions. For example, LBP can regulate the composition of intestinal microbes and the richness and diversity of species in order to help digest or resist ANFs or other inflammatory factors in SM that the body cannot process (Cao et al., 2021). A growing number of studies have shown that SCFAs and lactic acid produced by polysaccharide fermentation can lead to a decrease in intestinal pH, thus inhibiting the growth of pathogenic bacteria and promoting the proliferation of beneficial bacteria (Hu et al., 2014; Ma et al., 2017). Therefore, the fermentation of LBP in the intestinal tract may be one of the reasons for the changes in the composition of the intestinal microbiota. The addition of high SM in the diet not only caused the up-regulation of soybean saponin metabolites but also decreased the metabolites amphibine H and L-rhamnose, which was an undesirable effect of SM. Interestingly, this down-regulation was well compensated for by the addition of LBP, with significantly up-regulated metabolites amphibine H and L-rhamnose. In addition, studies have shown that LBP can promote the adhesion of lactobacillus to damaged Caco-2 cells, which indicates that LBP may repair the intestinal mucosal barrier by affecting intestinal microorganisms (Liu et al., 2022). Lu et al. (2021) reported that LBP activates intestinal epithelial cells to enhance the intestinal mucosal barrier. Therefore, on the one hand, LBP can improve the thickness of the intestinal mucosa by regulating intestinal microbial metabolites and repairing the intestinal mucosal barrier through microbial adhesion, thus reducing SBMIE, which is fully reflected in the results of enzyme activity index, tissue morphology observation, and metabolite analysis in this experiment. On the other hand, the intestinal epithelial cells may also directly utilize LBP to promote their development and maturation, thereby repairing the damaged intestinal mucosal barrier and reducing the invasion of ANFs in SM.

Naturally, this study focuses more on genetic aspects related to inflammatory factors and tight junction protein expression. IL-1β is a potent pro-inflammatory cytokine that plays a central role in intestinal inflammation and can lead to increased intestinal tight junction permeability when it reaches a certain concentration (Al-Sadi, 2007). Similarly, the pro-inflammatory cytokines IL-17, IL-22, and IL-26 also play a role in intestinal inflammation; IL-17 can mediate host defense against extracellular pathogens, and the continuous secretion of the IL-17 protein is also an important part of inflammation (Úsuga-Úsuga et al., 2022). Both IL-22 and IL-26 belong to the IL-10 family of cytokines, and IL-22 plays an important role in promoting inflammation, the production of antimicrobial peptides, and tissue repair on the barrier surface. The specific effect depends on the cell and cytokine environment (Takahashi et al., 2011; Yan et al., 2021). IL-26 is a novel mediator that overexpresses inflammation in activated or transformed T cells, which may be both a driver and an effector of inflammation, ultimately leading to persistent inflammation (Larochette et al., 2019). According to our results, the addition of LBP inhibited the expression of pro-inflammatory factors such as *IL-1β*, *IL-17*, *IL-22*, and *IL-26*, while promoting the expression of the tight junction protein-related gene *Occludin*. The expression of proinflammatory factors is directly related to the occurrence of intestinal inflammation, and reducing the expression of proinflammatory factor genes can significantly prevent the occurrence of inflammation. Moreover, the increase in *Occludin* gene expression can repair the intestinal mucosal barrier and reduce intestinal mucosal permeability, thus alleviating intestinal inflammation. It is obvious that LBP has a beneficial effect on relieving intestinal inflammation. Interestingly, however, we found that LBP may have a preference for regulating pro-inflammatory factors, with no significant effect on anti-inflammatory factors. At the same time, numerous reports have confirmed our conjecture (Cui et al., 2020; Du et al., 2016; Sun et al., 2022).

In regards to mechanism of action, our data suggest that LBP alleviates SM enteritis via the MAPK and Toll-like receptor (TLR) signaling pathway, and inhibits the expression of important genes *MAP* and *NF-κB2* in the MAPK signaling pathway in response to the inflammatory alleviating effect of LBP. The transcription factor NF-κB acts as a master switch regulating many pro-inflammatory factors (Hayden and Ghosh, 2011). The down-regulation of its gene is undoubtedly attributable to LBP, and the down-regulation of the above-mentioned pro-inflammatory factor genes may also be related to the down-regulation of the *NF-κB* gene. TLR2 belongs to the TLR family, and its signal transduction can promote the production of pro-inflammatory factors (Wetzler, 2003). Therefore, LBP may inhibit the production of pro-inflammatory factors by regulating the MAPK signaling pathway and the TLR signaling pathway, thus alleviating SBMIE. Similarly, many other studies have reported that LBP reduces the body's inflammatory response through NF-κB and MAPK signaling pathways (Qi et al., 2022; Sun et al., 2023; Jiang et al., 2023). Furthermore, there was a substantial intake of SM in the diet, drug metabolism-cytochrome P450, metabolism of xenobiotics by cytochrome P450, glycine, serine, and threonine metabolism, and arachidonic acid metabolism, and significant changes were observed in all of them. Morgan (2009) published a similar report revealing that downregulation of cytochrome P450s and other drug-metabolizing enzymes are linked to inflammation. The enrichment of two cytochrome P450-related pathways may indicate that it reduces the “toxicity” of SM and alleviates the adverse effects of SM through a series of metabolic pathways. According to our data, when LBP was added to a high-soy meal diet, the metabolic pathway changes were concentrated in glycine, serine and threonine metabolism, drug metabolism-other enzymes, and drug metabolism-cytochrome P450. This indicates that LBP may not be able to change the metabolic pathway of the body in response to a large amount of SM, but it can act on the above metabolic pathways to strengthen or weaken them to enhance the body's metabolism of SM and reduce the adverse effects of SM. Changes in metabolites also suggest this. As mentioned before, the addition of SM up-regulated the metabolites soyasaponin IV and soyasapogenol C, and down-regulated amphibine H, L-rhamnose, and folate; however, the addition of LBP resulted in changes in metabolites in the 0% SM group, including the up-regulation of metabolites amphibine H and L-rhamnose, and the down-regulation of taurodehydrocholic acid. The results showed that the addition of SM increased the secretion of saponin metabolites in spotted sea bass. However, SM down-regulated the metabolites amphibine H, L-rhamnose, and folate, and LBP up-regulated them accordingly. L-rhamnose is a monosaccharide widely found in plants and an important component of the cell wall of some microorganisms (Southard et al., 1959). In this study, although LBP contains L-rhamnose, the amount of LBP added to the experimental diets was very small, so changes in L-rhamnose were not observed as a result of direct digestion of LBP in the intestinal tract of spotted sea bass. In addition, oligosaccharide fragments released by LBP during microbial degradation may be utilized by other species that cannot utilize polysaccharides alone, thus forming an ecological network of polysaccharide utilization among symbionts in the gut, which makes active oligosaccharide fragments of LBP difficult to obtain (Rakoff-Nahoum et al., 2014). However, the oligosaccharide metabolites observed in this study indicated that the intestinal microbial composition of sea bass was dominated by species that could degrade polysaccharides, and few microorganisms can use polysaccharides alone.

In this study, the effect of LBP on alleviating SM enteritis provides strong evidence to recommend its application in the aquaculture industry, providing more choices for the safe aquaculture of aquatic products, and providing a theoretical basis for the sustainable feed practice of replacing fish meal with renewable plant protein. This study only evaluated the effect of LBP on SM-type intestinal inflammation, but whether it can maintain such effects on IBD remains to be explored. At the same time, LBP, the interaction relationship and mechanisms between intestinal microbes and the intestinal tract are also interesting directions, and further studies on the SCFA metabolism of intestinal microbes, intestinal targeted metabolism, and quantitative studies on intestinal microbes are warranted. In addition, the negative effects of LBP are rarely reported. These potential negative effects not only affect the optimal use of polysaccharides, but also restrict the wide application of polysaccharides in other industries. Therefore, it is necessary to report the negative effects of polysaccharides in subsequent studies.

## 5 Conclusions

LBP can reduce intestinal mucosal permeability, change intestinal microbial structure, and increase species richness and diversity, so as to repair the damage caused by high SM content in the intestinal tract of spotted sea bass. On the other hand, by affecting MAPK, the TLR signaling pathway, and drug metabolism-cytochrome P450, LBP can down-regulate genes related to proinflammatory factors, up-regulate genes related to tight junction proteins, reduce saponin metabolites, and increase the expression of amino acid metabolites to reduce intestinal inflammation. These results reveal the potential of LBP in alleviating SBMIE in fish and provide a theoretical basis for alleviating FM shortages and realizing green and sustainable healthy farming.

## CRediT authorship contribution statement

**Longhui Liu and Zhongbao Li** conceived and designed the experiments. **Longhui Liu and Zhangfan Huang** performed the experiments. **Longhui Liu, Zhangfan Huang, Zhongying Long, Huihui Qin, Yanbo Zhao, Hao Lin, Sishun Zhou, Jianrong Ma, and Lumin Kong** participated in the sample collection. **Longhui Liu** performed the analyses. **Longhui Liu** analyzed the data, wrote the paper, and prepared figures and tables. All authors discussed the results together. **Zhongbao Li** reviewed drafts of the paper. All authors have read and approved this version of the article, and due care has been taken to ensure the integrity of the work.

## Data availability statement

The data that support the findings of “3.5 intestinal transcriptome analysis and RT-qPCR” are available in Mendeley Data: Liu, Longhui (2023), “Evaluation of the effect of *L. barbarum* polysaccharides in alleviating soybean meal induced enteritis of spotted sea bass *L. maculatus*”, Mendeley Data, V1, doi: 10.17632/kk3fg8zcyf.1.

## Declaration of competing interest

We declare that we have no financial and personal relationships with other people or organizations that can inappropriately influence our work, and there is no professional or other personal interest of any nature or kind in any product, service and/or company that could be construed as influencing the content of this paper.
